# A transcriptional roadblock protects yeast centromeres

**DOI:** 10.1093/nar/gkac117

**Published:** 2022-03-07

**Authors:** Sabrine Hedouin, Glennis A Logsdon, Jason G Underwood, Sue Biggins

**Affiliations:** Howard Hughes Medical Institute, Basic Sciences Division, Fred Hutchinson Cancer Research Center, Seattle, WA 98109, USA; Department of Genome Sciences, University of Washington School of Medicine, Seattle, WA 98195, USA; Pacific Biosciences (PacBio) of California, Incorporated, Menlo Park, CA 94025, USA; Howard Hughes Medical Institute, Basic Sciences Division, Fred Hutchinson Cancer Research Center, Seattle, WA 98109, USA

## Abstract

Centromeres are the chromosomal loci essential for faithful chromosome segregation during cell division. Although centromeres are transcribed and produce non-coding RNAs (cenRNAs) that affect centromere function, we still lack a mechanistic understanding of how centromere transcription is regulated. Here, using a targeted RNA isoform sequencing approach, we identified the transcriptional landscape at and surrounding all centromeres in budding yeast. Overall, cenRNAs are derived from transcription readthrough of pericentromeric regions but rarely span the entire centromere and are a complex mixture of molecules that are heterogeneous in abundance, orientation, and sequence. While most pericentromeres are transcribed throughout the cell cycle, centromere accessibility to the transcription machinery is restricted to S-phase. This temporal restriction is dependent on Cbf1, a centromere-binding transcription factor, that we demonstrate acts locally as a transcriptional roadblock. Cbf1 deletion leads to an accumulation of cenRNAs at all phases of the cell cycle which correlates with increased chromosome mis-segregation that is partially rescued when the roadblock activity is restored. We propose that a Cbf1-mediated transcriptional roadblock protects yeast centromeres from untimely transcription to ensure genomic stability.

## INTRODUCTION

Centromeres are specialized regions of eukaryotic chromosomes essential for the faithful segregation of the genetic material during cell division. They serve as the specific site of assembly of the kinetochore, a highly conserved macromolecular complex that links chromosomes to dynamic spindle microtubules (1,2). Despite their essential function, centromeric sequences are poorly conserved across species, often enriched in highly repetitive DNA, forming regional centromeres that can span several megabases and are flanked by constitutive pericentromeric heterochromatin domains (3). Given the lack of apparent genetic determinant in most species, centromere identity is primarily defined epigenetically through a unique and conserved chromatin architecture, largely characterized by the specific incorporation of a centromeric histone H3 variant called Centromere protein-A (CENP-A) (4,5). CENP-A containing nucleosomes are necessary and sufficient for the hierarchical and conserved recruitment of the inner kinetochore complexes, which in turn promote the assembly of outer kinetochore complexes that mediate microtubule attachment (1).

Although it was originally thought that centromeres are transcriptionally inert domains, it was recently found that they are subject to a conserved low-level of non-coding transcription, adding further complexity to the current model of the organization and regulation of centromere function. Although these centromere-derived RNAs (cenRNAs) differ greatly in size and sequence, altering their levels has detrimental effects on kinetochore function and promotes chromosome instability (6–15), suggesting that transcription must be tightly regulated. CenRNAs and centromeric transcription have recently been implicated in kinetochore assembly and maintenance as well as regulation of kinetochore-microtubule attachments; however, their precise molecular role in these functions is still not clear owing to the difficulty in manipulating them *in vivo* (8,10,13,16–22). Likewise, very little is known regarding the exact nature of these RNAs and the factors that regulate transcription of this locus. These questions have been difficult to address directly in most eukaryotes due to the technical difficulties inherent in the study of highly repeated sequences and the lack of methodologies to purify or assemble functional kinetochores to directly test their function.

The budding yeast *Saccharomyces cerevisiae* point centromeres represent an ideal model to study centromere transcription due to their simple organization and lack of complex pericentromeric heterochromatin domains. Each of the sixteen centromeres (CENs) are genetically specified by a similar but not identical ∼120bp sequence, consisting of three conserved centromere DNA elements (CDEI-II-III) around which a single CENP-A^Cse4^ nucleosome is assembled (23). CDEI is the binding site for Cbf1, a basal transcription factor with both repressor and activator activity, while CDEIII is bound by the CBF3 complex which promotes the recruitment and deposition of CENP-A^Cse4^ on the AT-rich CDEII element (24–28). Like other species, yeast centromeres were previously thought to be devoid of any endogenous transcriptional activity, and they were proposed to even have a transcription blocking ability (29). Consistent with this, forced transcription of a centromere leads to its inactivation (30), demonstrating that high levels of transcription are deleterious to point centromere function. Paradoxically, more recent studies showed that yeast centromeres undergo low levels of transcription and produce cenRNAs that promote point centromere function, as their targeted degradation impairs chromosome segregation (9,31,32). CenRNAs are detected most easily in S phase, which correlates with the transient disassembly and reassembly of the kinetochore (33). Cbf1 was shown to negatively regulate centromere transcription, as its deletion leads to constitutive transcription throughout the cell cycle, which correlates with impaired binding of some kinetochore proteins and increased rates of chromosome mis-segregation (9,31). This apparent dichotomy regarding centromere transcription suggests that specific mechanisms might be at play to both protect centromeres from surrounding transcription as well as to allow their timely transcription. However, the molecular details of this process remain unclear because we still lack a comprehensive understanding of the transcriptional landscape at centromeres during the cell cycle.

Here, we performed long-read RNA isoform sequencing (Iso-Seq) combined with transcript enrichment to identify the full repertoire of cenRNAs in budding yeast. We found that transcription at and around centromeres is complex and heterogenous between chromosomes and highly regulated during the cell cycle. Transcription initiates in the pericentromeric domain and usually terminates before entering the centromere, making cenRNAs a small subset of the surrounding RNA repertoire. While most pericentromeres are transcribed throughout the cell cycle, centromeres only become accessible to the transcription machinery in S phase. We found that centromere accessibility is regulated via a transcriptional roadblock mechanism mediated by Cbf1. Restoring the roadblock activity at CDEI with another transcription factor partially rescues chromosome segregation defects, suggesting that one activity of Cbf1 at centromeres is to safeguard this domain from surrounding transcription. Overall, our findings highlight the importance of shielding centromeres from transcription during most cell cycle phases to protect kinetochore function and ensure genomic stability.

## MATERIALS AND METHODS

### Strain construction and microbial techniques

The *S. cerevisiae* strains used in this study are listed in Supplemental Table S1 and are derivative of SBY3 (W303). All liquid cultures were grown in yeast peptone dextrose rich (YPD) media. Standard genetic crosses, media and microbial techniques were used (34). Gene deletions, and epitope tagged alleles (3Flag, 13myc, and 3V5) were constructed at the endogenous loci by standard PCR-based integration as described in (35) and confirmed by PCR. Plasmid mutagenesis was performed as described (36). Genome editing of *CEN8* was performed as described in (37). Briefly, strain SBY5082 was transformed with 500 ng of plasmid pSB3303 and 2 μg of gBlock repair template SB6996 (for CDEI mutant) or SB6997 (for Reb1-BS replacement). Transformed colonies were counter-selected on 5-Fluoroorotic Acid plates to ensure loss of the Cas9 plasmid. The *CEN8* locus was PCR amplified and sequenced to verify the successful editing and correct transformants were backcrossed. The plasmids and primers used to generate strains are listed in Supplemental Tables S2 and S3.

### Centromeric probe design

To enrich for full-length centromeric cDNA molecules, we designed 48 5′-biotinylated oligonucleotide probes against the *S. cerevisiae* S288C strain's CDEs and flanking regions (three probes per centromere). Each probe was 103–120 bases long (mode = 120 bases) and designed to have a similar GC content (10.8–37.8%; mode = 25.8%) and melting temperature (66.9–70.8°C; mode = 67.6°C). The probes were ordered from IDT as a pool of xGen Lockdown probes and stored at –20°C. The name, sequence, strand origin, genomic coordinate, GC content, and melting temperature of each probe is listed in Supplemental Table S4.

### RNA preparation, cDNA synthesis and hybridization capture of cDNA molecules

WT (SBY3) and *cbf1Δ* (SBY4958) cells were grown in yeast peptone dextrose rich (YPD) media and arrested in G1 by adding 1 μg/ml α-factor to early log phase cells. When arrest was complete (∼3 h), cells were washed twice with an equivalent volume of YPD and released into medium lacking the α-factor pheromone. ∼75 min after G1 release, 1 μg/ml α-factor was added to prevent a second cell division. Samples were collected every 20 min after release, washed with H_2_O and flash frozen in liquid nitrogen. Cells harvested just after α-factor release form the G1 fraction (*t* = 0).

RNA was extracted using TRIzol (Thermo Fisher, #15596) following the manufacturer's instructions. Briefly, frozen cell pellets were resuspended in TRIzol and lysed using glass beads and a bead beater (Biospec products). RNA was extracted twice with chloroform, precipitated with isopropanol, washed twice with 75% ethanol and resuspended in H_2_O. RNA was stored at –80°C. Cell cycle progression was monitored by RT-PCR analysis, as described below, of *HTA1* (histone H2A) and *SWI5* genes whose expression peaks in S phase and G2/M respectively. RNAs from *t* = 0 min (G1 phase) and *t* = 40 min (S phase) were selected for further polyadenylated tail enrichment and sequencing.

Polyadenylated (poly(A)) RNA was enriched using Oligo d(T)25 Magnetic Beads (NEB, #S1419S) following the manufacturer's instructions, using ∼150 μg of purified total RNA.

Poly(A)-enriched RNA was treated with DNase I to remove any contaminating DNA using the RNA Clean & Concentrator kit (Zymo, #R1014) according to the manufacturer's instructions. RNA was ligated to the universal miRNA cloning linker (NEB, #S1315S) by combining 100 ng of poly(A)-enriched RNA with 1 μM universal miRNA cloning linker, 1X T4 RNA ligase buffer, 20% PEG 8000, 100 U T4 RNA ligase 2 (truncated KQ) (NEB, #M0373S), and 10 U SUPERase-In RNase inhibitor (ThermoFisher Scientific, #AM2694) in a 20 μl volume and incubating at 25°C for 2 h in a thermocycler. The linker-ligated RNA was purified using the RNA Clean & Concentrator kit (Zymo, #R1014) and eluted in 15 μl RNase/DNase-free water. The RNA was reverse-transcribed into double-stranded cDNA using the SMARTer PCR cDNA Synthesis Kit (Clontech, #634925) with modifications to the manufacturer's protocol. Briefly, 10–50 ng of RNA was mixed with 1 mM dNTP mix and 1 μM of the following oligo: 5′- AAGCAGTGGTATCAACGCAGAGTNNNNNNATTGATGGTGCCTACAG-3′, where N is either an A, C, G or T, and the string of Ns creates a unique molecular identifier (UMI) that serves as a barcode for each cDNA molecule. This mixture was heated at 65°C for 2 min on a thermocycler and then combined with a pre-heated mixture of 1× First-Strand Buffer, 10 μM DTT, 1.2 μM Clontech SMARTer IIA template-switch oligo, 5 U SUPERase-In RNase inhibitor, and 100 U Maxima H Minus Reverse Transcriptase (ThermoFisher Scientific, #EP0751). This 10 μl mixture was incubated at 50°C for 1 h, 45°C for 30 min, and 85°C for 5 min before being cooled on ice. The resulting double-stranded cDNA was purified using the Monarch PCR & DNA Cleanup Kit (NEB, #T1030S) and amplified via PCR with 1× KAPA HiFi HotStart ReadyMix (Roche, #KR0370) and 1 μM of the following oligo: 5′-AAGCAGTGGTATCAACGCAGAGT-3′ for 12 PCR cycles. The amplified DNA was purified with the QIAquick PCR Purification Kit (Qiagen, #28104) and eluted in 30 μl Elution Buffer.

To capture centromeric cDNA molecules, the xGen Hybridization and Wash Kit (IDT, #1080577) was used with some modifications to the manufacturer's protocol. Briefly, 500 ng–1 μg of double-stranded cDNA was lyophilized with 6 μM 5′ blocking oligo (Clontech SMARTer IIA template-switch oligo), 6 μM 3′ blocking oligo (Pacbio 3′ blocker-16bpBC), and 1 μg salmon sperm (Invitrogen, #15-632-011) using a SpeedVac™ system. The lyophilized DNA was resuspended in 17 μl hybridization mastermix consisting of 8.5 μl xGen 2X Hybrization Buffer, 2.7 μl xGen Hybridization Buffer Enhancer, 1.8 μl water, and 4 μl of 0.75 μM biotinylated probes. The solution was heated at 80°C for 5 min, incubated at 95°C for 1 min, and then incubated at 65°C for 2–4 h. Washed streptavidin beads (Invitrogen, #65306) were added to the mixture and incubated at 65°C for 45 min. The beads were washed with a series of heated and room temperature washes according to the manufacturer's protocol. The remaining DNA on the beads were amplified via PCR with 1× KAPA HiFi HotStart ReadyMix (Roche, #KR0370) and 1 μM of the following oligo: 5′-AAGCAGTGGTATCAACGCAGAGT-3′ for 15 PCR cycles according to the manufacturer's instructions. The resulting DNA was purified and checked via Agilent's BioAnalyzer System to ensure successful amplification.

### SMRTbell library preparation and circular consensus sequencing (CCS)

Sequencing libraries were prepared using the SMRTbell Express Template Prep Kit 2.0 (Pacific Biosciences, #100-938-900) according to the manufacturer's protocol. Briefly, 250 ng of DNA was damage-repaired by mixing it with 7 μl DNA prep buffer, 0.6 μl NAD, and 2 μl DNA Damage Repair Mix v2. This reaction was incubated at 37°C for 30 min before proceeding to the end-repair step. For the end-repair step, 3 μl End Prep Mix was added to the reaction and incubated at 20°C for 30 min, followed by a second incubation at 65°C for 30 min. The reaction was then mixed with 3 μl barcoded overhang adapter, 30 μl Ligation Mix, 1 μl Ligation Enhancer, and 1 μl Ligation Additive to ligate the barcoded adapter. This reaction was incubated at 20°C for 1 h. The SMRTbell libraries were cleaned up with ProNex beads (Promega, #NG2001), pooled, and sequenced on a SMRT Cell 8M on the Pacific Biosciences Sequel II in CCS mode with the following parameters: –noPolish –minLength 10 –minPasses = 3).

### Sequencing data processing

All CCS reads were demultiplexed and trimmed to remove the adapter with Lima v1.10.0 (https://lima.how/) and the following parameters: –isoseq –dump-clips. The resulting reads were tagged for deduplication and trimmed to remove the linker and UMI with the isoseq3 v3.2.2 (https://github.com/PacificBiosciences/IsoSeq) tag step and the following parameters: –design T-17B-7U. Full-length non-chimeric (FLNC) reads were extracted with the isoseq3 v3.2.2 refine step and the following parameters: –min-polya-length 10 –require-polya. The resulting FLNC reads were deduplicated with the isoseq3 v3.2.2 dedup step and the following parameters: –max-tag-mismatches 0 –max-tag-shift 0. The resulting FLNC, deduplicated reads were mapped to the April 2011 sacCer3 yeast reference genome via Minimap2 v2.17-r941 (38) with the following parameters: -ax splice -t {threads} -G2k -uf -C5 {ref.fasta} {input.fastq}. SAM files were filtered using SAMtools (39) with FLAG score 2308 to prevent multi-mapping of reads, and BAMs were visualized in the Integrated Genome Browser (IGB; v9.1.8). The number of reads entering, spanning, or flanking the CDEs on each chromosome were quantified using BEDtools v2.25.0 (40) by intersecting the alignment file with a BED file of each CDE region and were normalized to the read depth of each library. (peri)CEN transcripts were characterized as transcripts which termination occurred within 50 bp of each CEN while CEN transcripts were characterized as transcripts entering the CEN by at least 1 bp. The 50 bp cut-off was selected to minimize background from adjacent genic transcripts. Data presented in the manuscript represent the average between the two replicates. Iso-Seq profiles presented in the manuscript are from the first replicate. Similar results were observed for the second replicate and therefore not shown for clarity of visualization.

To characterize the origin of (peri)cenRNA transcription, we analyzed the position of the main transcription start site relative to the closest neighboring gene and to the position of published mapped canonical nucleosomes (41). We considered a Nucleosome Depleted Region (NDR) to be a region >80 bp of spacing between two adjacent nucleosomes.

### Analysis of gene expression

RNA from early log phase cultures or from G1 arrest/release kinetics was extracted using TRIzol (Thermo Fisher, #15596) as described above. Contaminant genomic DNA was eliminated using TURBO DNA-free kit (Ambion, #AM1907). RNA concentration was measured on a Nanodrop™ (Thermo Fisher). 1 μg of DNase-treated RNA was reverse transcribed using RevertAid Reverse Transcriptase (ThermoFisher Scientific, #EP0442) in a 20 μl reaction using 1.25 ng/μl random hexamer and 2.5 μM oligo(dT)_18_ primers for 1 h at 42°C and analyzed by PCR using Phusion High Fidelity DNA Polymerase (ThermoFisher Scientific, #F530S), or qPCR using PowerUp™ SYBR™ Green Master Mix (Thermo Fisher, #A25742) or Forget-Me-Not EvaGreen qPCR Master Mix (Biotium, #31045) with primers listed in Supplemental Table S3. qPCR was performed using a Quantstudio™ 5 Real-Time PCR System (Applied Biosystem). A final dissociation stage was performed to verify the specificity of the PCR primers. Primer efficiency of each primer pair was evaluated by standard curves with 2-fold serial dilutions of cDNA and is specified in Supplemental Table S3. The relative expression of the (peri)cenRNAs was normalized to *UBC6* expression, which is stably expressed throughout the cell cycle. The relative fold changes were calculated using the ΔΔCt method (42).

To demonstrate that cenRNAs are polyadenylated, total RNAs were fractionated into poly(A)+ and poly(A)- fractions. Poly(A)+ RNAs were enriched using Oligo d(T)25 Magnetic Beads (NEB, #S1419S) following the manufacturer's instructions. The supernatant left after the initial binding of the beads was saved, and RNAs were purified using acid phenol extraction and ethanol precipitation, forming the poly(A)– fraction. RNAs from total, poly(A)+ and poly(A)– fractions were DNase treated and 250 ng of RNA was reverse transcribed using only random hexamer primers. *COX3*, a mitochondrial mRNA without poly(A) tail, was used as a control for the poly(A)– fraction (43).

### *De novo* kinetochore assembly assay and T7 *in vitro* transcription

Preparation of DNA templates for the kinetochore assembly assay were performed as described in (44) with a few modifications. In order to initiate T7 transcription, the T7 promoter sequence was added to the 5′ non-biotinylated oligo used to amplify the DNA templates. Plasmids and primers used to generate the DNA templates are listed in Supplemental Tables S2 and S3. M280 streptavidin Dynabeads (Invitrogen, #11205D) were coated with a 10:1 ratio of *CEN3* to *ampC* biotinylated DNA. De novo kinetochore assembly was performed with whole cell extract from SBY19103 (*CBF1-3FLAG*, *REB1-13MYC*, *CNN1-3V5*) as described in (44). Briefly, 500 μl of whole cell extract and 20 μl of DNA coated M280 Dynabeads were incubated at room temperature for 90 min to allow kinetochore assembly. Following the last wash, beads were resuspended in 40 μl of Buffer L ((25 mM HEPES pH 7.6, 2 mM MgCl_2_, 0.1 mM EDTA pH 7.6, 0.5 mM EGTA pH 7.6, 0.1% NP-40, 175 mM K-glutamate and 15% glycerol) supplemented with 0.1% BSA, 10 U of DraI (NEB, #R0129S) and 2.5 U of SspI (NEB, #R0132S) and incubated at 30°C for 15 min to allow the cleavage of kinetochore-unbound DNA. Beads were then washed once with Buffer L, split into two tubes and resuspended in 20 μl of Buffer L supplemented with 0.5 mM rNTP mix (NEB, #N0466S), 2 μM spermidine (Sigma, #S2501) and 20 U of RNase inhibitor murine (NEB, #M0314L). One tube had an addition of 100 U of T7 RNA Polymerase (NEB, #M0251L). Both tubes were incubated at 30°C for 30 min. The supernatant containing *in vitro* transcribed RNAs was collected and RNAs were purified by acid phenol extraction and ethanol precipitation. RNA pellet was resuspended in 20 μl of water and 3 μl was run on a 6% Novex™ TBE–urea gel (Invitrogen, #EC68652BOX) in 1× TBE buffer. Gel was stained with SYBR Gold (Invitrogen, #S11494) and imaged on a ChemiDoc™ MP imaging system (Biorad). Band intensity was analyzed using ImageJ software (v1.53f51).

### Chromosome segregation assay

Cells were grown in YPD medium. Exponentially growing *MATa* cells also carrying a tandem array of lacO sequences integrated proximal to *CEN8* (45) and a LacI-GFP fusion (46,47) were arrested in G1 with 1 μg/ml α-factor. When arrest was complete, cells were released into medium lacking α-factor pheromone but supplemented with 200 μM CuSO_4_ to induce the expression of the LacI-GFP fusion. ∼75 min after G1 release, 1 μg/ml α-factor was added to prevent a second cell division. Samples were taken ∼100 min after G1 release to quantify chromosome segregation in anaphase. Samples were fixed with 3.7% formaldehyde (Fisher chemical, #252549) in 0.1 M potassium phosphate (KPi) buffer (pH 6.4) for 10 min, washed once with 0.1 M KPi pH 6.4 and resuspended in 1.2 M Sorbitol, 1% Triton X-100, 0.1 M KPi pH 7.5 supplemented with 2 μg/ml DAPI. Cells were plated on 0.5 mg/ml Concanavalin A (Sigma, #L7647) coated coverslips and visualized on a Deltavision™ Ultra equipped with a 100×/1.40 PlanApo N oil-immersion objective (Olympus). Two hundred cells were counted per genotype and per experiment (*n* = 3).

### Statistical analysis

Statistical analyses were performed with GraphPad Prism software version 8.4 (GraphPad Software, San Diego, CA, USA). Each test is specifically identified in the figure legends. Note that we could not perform a statistical analysis to test the significance of differences observed between the number of counts per thousand (CPT) reads between samples. This is because the hybridization capture enrichment step introduces a bias in the read distribution that prevents the application of common bioinformatics tools that calculate differential ‘gene’ expression between samples with an associated *P*-value.

## RESULTS

### Characterization of the transcriptional landscape around centromeres

**Figure 1.**
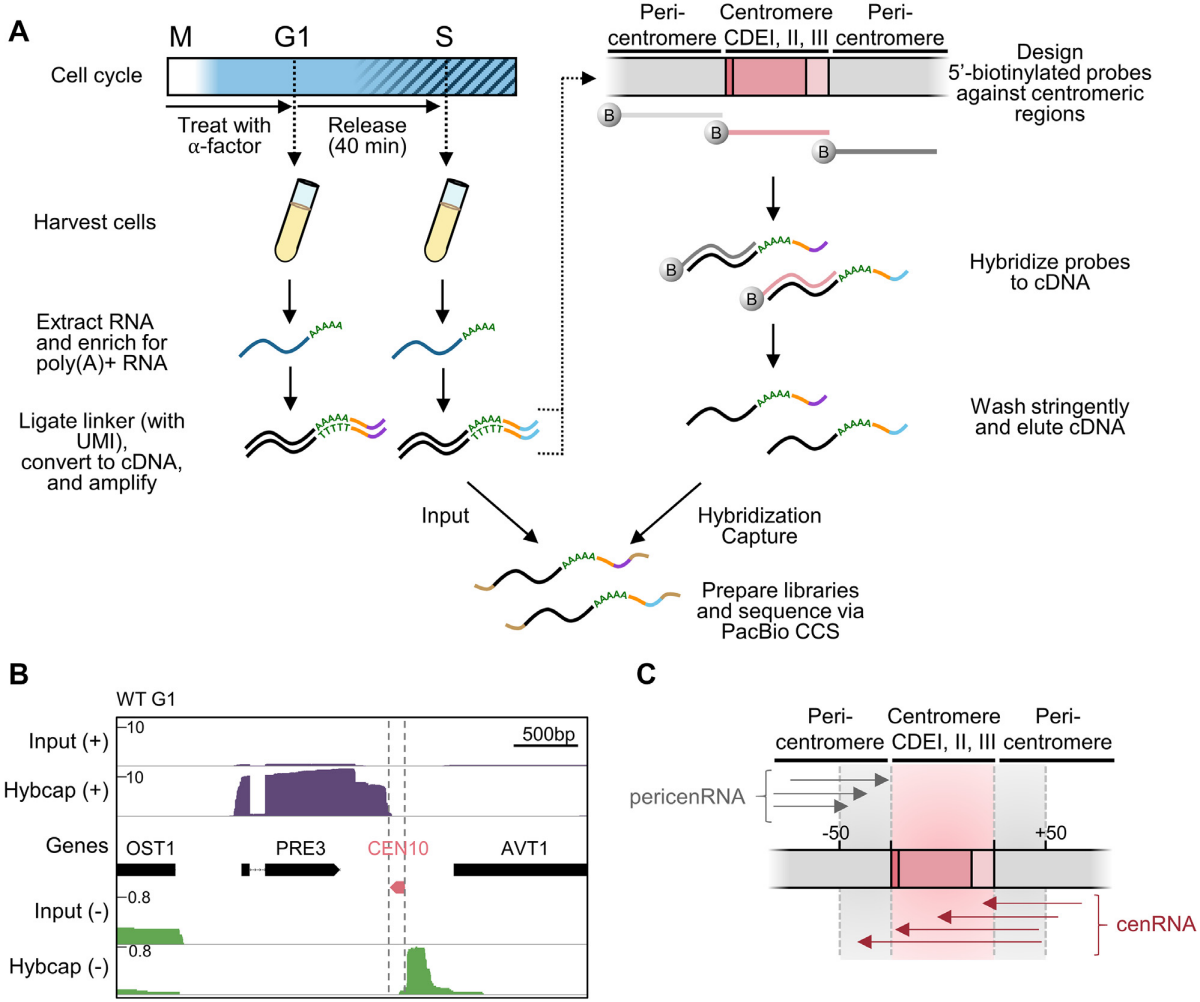
Hybridization capture and long-read sequencing of lowly expressed cenRNAs. (**A**) Schematic illustrating the procedure to capture and sequence RNA molecules generated at or near budding yeast centromeres. Cells were synchronized in G1 phase with α-factor and then released into the cell cycle. Cells were harvested immediately after release (G1 phase) and 40 min after release (S phase). RNA (dark blue) was extracted and enriched for those that were polyadenylated. Poly(A)+ RNA was ligated to a linker (orange) containing a unique molecular identifier (UMI; purple or light blue). The RNA was next reverse transcribed into double-stranded cDNA (black) and amplified via PCR. The cDNA was hybridized to 5′-biotinylated DNA probes (dark grey, light grey and pink) designed against the three CDEs (pink) of all 16 centromeres as well as the flanking regions (gray) to enrich for centromeric and pericentromeric RNAs. The probes were washed stringently and the remaining cDNA was eluted from the probes. Both input and enriched (Hybcap) cDNAs were prepared into a library by adding 5′ and 3′ barcoded adapters (brown) and sequenced via Pacific Biosciences (PacBio) long-read circular-consensus sequencing (CCS). (**B**) Iso-Seq profiles of WT G1 cells before (Input) and after hybridization capture (Hybcap). Position of centromere boundaries is shown by the vertical grey dotted lines. Reads coming from the (+) strand are shown in purple while reads coming from the (–) strand are shown in green. (**C**) Schematic illustrating the nomenclature used throughout the paper. PericenRNAs correspond to transcripts converging towards the CEN but stopping within 50 bp of the CEN border (grey arrows) while cenRNAs correspond to transcripts that converge towards the CEN and enter it by at least 1bp (dark pink arrows). cenRNAs are pericenRNAs that readthrough the CEN.

Due to their very low abundance, cenRNAs are usually absent from published short read RNA-seq datasets, hindering the identification of their initiation and termination sites that would facilitate their manipulation. Therefore, to better understand the regulation and function of yeast centromere transcription, we sought to elucidate the full extent of the cenRNA landscape. To do this, we took advantage of long-read RNA isoform sequencing (Iso-Seq) technologies that allow determination of full-length transcript sequence (48,49). Centromere transcription is highest during S phase (9,31), so we prepared RNAs from both G1 and S phase populations for comparison. Because cenRNAs are polyadenylated (9) (Supplemental Figure S1), we first purified poly(A)+ RNAs. To enrich for cenRNAs that would have escaped detection by other methods, we further enriched for cenRNA cDNAs by hybridization capture. We followed a previously established protocol with two modifications (50) (Figure 1A). First, to avoid any bias introduced during reverse transcription from the AT rich CDEII element, we ligated an adapter to the 3′ end of the poly(A) tail and used it to initiate the first strand of reverse transcription instead of using oligodT primers. Secondly, to maximize the enrichment of cenRNA molecules during the hybridization capture step, we designed three 105–120 nt biotinylated probes specific to each centromere: one probe complementary to the core centromere and two probes complementary to each of the flanking pericentromeric regions (48 unique probes total, Figure 1A and Supplemental Table S4). The libraries were then sequenced via Pacific Biosciences (PacBio) circular-consensus-sequencing (CCS) and aligned to the yeast reference genome. Two independent replicates were performed for each time point and showed high correlation (*r*² ≥ 0.94) (Supplemental Figure S2A).

### Iso-Seq uncovers complex transcriptional profiles around centromeres

We initially verified that the input reads mapped to full-length ORFs and that we could detect known RNA isoforms that bear alternative polyadenylation cleavage sites, suggesting that we were successful in sequencing complete RNA isoforms (Supplemental Figure S2B) (51). We further checked that we successfully enriched for transcripts overlapping with the probes used for the hybridization capture (Figure 1B). Indeed, reads mapping to pericentromeres represented only ∼0.04% of input libraries whereas they represented ∼75% of the libraries following hybridization capture (∼1700-fold enrichment). In order to differentiate between centromeric and pericentromeric transcripts, we arbitrarily defined cenRNAs as any transcripts that enter the centromere by at least one base pair while pericenRNAs refer to any transcripts that terminate within 50 bp of the centromere border, whether they are of genic origin or not (Figure 1C). Of note, the aforementioned transcripts are derived from transcription converging towards the centromere. Unexpectedly, we also observed transcription starting at or around centromeres and diverging from them. While this study mainly focuses on converging transcription, an additional analysis of the divergent transcription is presented in the accompanying Supplemental note.

**Figure 2.**
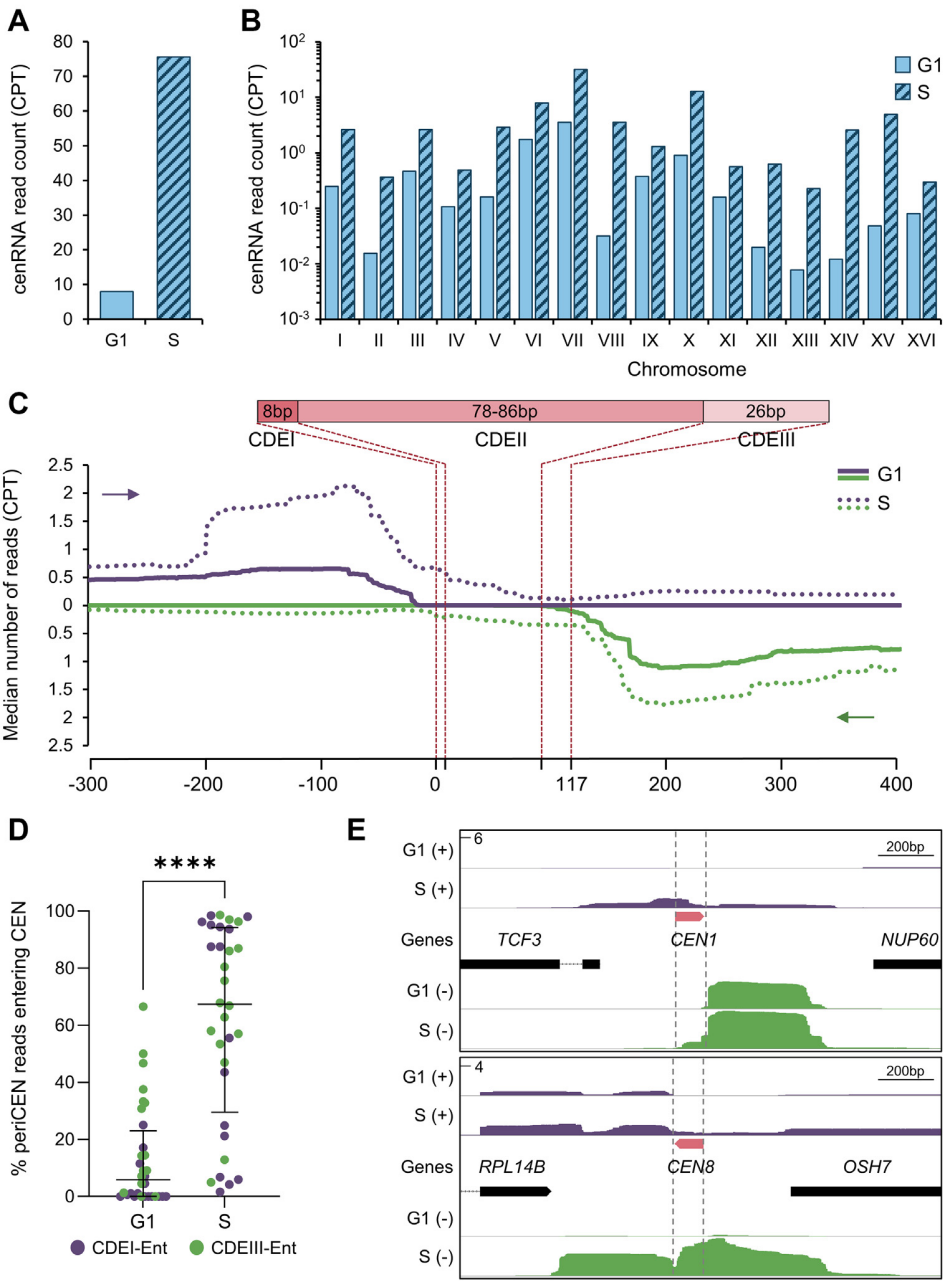
Iso-Seq reveals a complex transcriptional landscape at centromeres. (**A**) Number of normalized cenRNA reads (transcripts entering CEN by at least 1 nt) measured by counts per thousand (CPT) after hybridization capture enrichment. Input reads are not shown because they were not detected. (**B**) Distribution of normalized cenRNA read counts per thousand (CPT) per chromosome. (**C**) Aggregate plot of median read count around centromeres. All 16 centromeres, regardless of the strand of origin, were aligned at the beginning of the CDEI element (top plot) or the beginning of the CDEIII element (bottom plot). The 5′–3′ direction is indicated by a purple arrow for reads converging toward the CDEI element and a green arrow for reads converging towards the CDEIII element. The structure of the centromere is schematically shown on the top. (**D**) Distribution of the proportion of periCEN reads that enter the CEN in G1 versus S phase. Each centromere is represented by two dots for reads coming from each strand. Median plus interquartile range is displayed in black. *P*-values determined by a paired Wilcoxon test (****P < 0.0001). (**E**) Example of Iso-Seq profiles at *CEN1* and *CEN8*. Position of centromere boundaries is shown by the vertical grey dotted lines. Reads coming from the (+) strand are shown in purple while reads coming from the (–) strand are shown in green. The y-axis represents the maximum CPT.

Consistent with previous work, the reads overlapping with centromeres in the input samples are extremely rare and therefore not consistently detected (52,53). However, post-hybridization capture, we detected roughly 8 and 76 counts per thousand (CPT) of molecules overlapping centromeres, either from the + or – strand, in G1 and S respectively, confirming that the enrichment step was successful and that centromere transcription increases during S phase (Figure 2A). Due to the absence of reads in the input samples, we could not estimate the rate of cenRNA enrichment post-hybridization capture. Even after enrichment, cenRNAs transcripts represented only 0.79% of total sequenced reads in G1 and 7.56% in S phase (Supplemental Figure S3A). Surprisingly, most cenRNAs do not encompass the full centromere (all three CDEs) as they represented only 0.04% and 1.5% of total reads in G1 and S phase, respectively (Supplemental Figure S3A). This result likely explains why cenRNAs are hardly detectable by RT-PCR when primers designed to amplify the whole CEN are used (9,31,32,54), since they only represent a minor fraction of cenRNA isoforms.

We next analyzed the number of reads across all 16 centromeres. Although there was little detectable transcription at most centromeres in G1, some (like *CEN6* and *CEN7*) showed higher read counts (Figure 2B). During S phase, all 16 centromeres become more robustly transcribed, although levels still vary greatly between centromeres (Figure 2B). However, because the biotinylated probes used for cenRNA enrichment may vary in pull-down efficiency, the transcription levels between centromeres may not be accurately compared. Nonetheless, our data are consistent with published qPCR data where different cenRNAs varied in abundance (31).

We next sought to characterize the transcription start sites (TSS) of cenRNAs. As previously observed using rapid amplification of cDNA ends (RACE-PCR), they initiate from the pericentromeric domain (9). Interestingly, there is a high heterogeneity in how CEN transcription is initiated. Indeed, we categorized each TSS for all cenRNAs, whether they enter through the CDEI or CDEIII element, and identified 4 main ways through which transcription occurs: transcription readthrough from a neighboring gene, antisense initiation of a neighboring gene body, initiation in an intergenic region and finally, initiation using a nucleosome depleted region (NDR) as found in the promoter or the transcription termination site (TTS) of a neighboring gene (Table 1). Interestingly, the latter category is the source of the most abundant cenRNAs, and is consistent with properties of ncRNAs for which transcription often initiates in open chromatin region (55,56). Moreover, transcription initiation can also span hundreds of bases, suggesting that most cenRNAs lack a clearly defined promoter-like region and are likely products of pervasive transcription. Given this heterogeneity, the unique genomic context of each CEN likely dictates its transcriptional activity rather than depending on specific transcription factors. Indeed, while many putative binding sites for transcription factors are found in the vicinity of cenRNAs’ TSSs, none is present at all CENs and deletion of one candidate did not impair cenRNA levels (data not shown), leaving that possibility open but uncertain.

**Table 1.** Origin of transcription of (peri)cenRNAs. Classification of the transcriptional origin of (peri)cenRNAs for both strands, entering the CEN either through the CDEI element (CDEI-Ent) or through the CDEIII element (CDEIII-Ent), relative to their genomic environment. The most abundant isoform for each (peri)cenRNAs, as shown in Supplemental Figure S4C, is underlined. Convergent neighboring genes can have their transcription reading through the centromere and/or have their transcription termination site utilized as a transcription start site. A schematic of the four types of transcription initiation is shown at the top. NDR, Nucleosome Depleted Region.

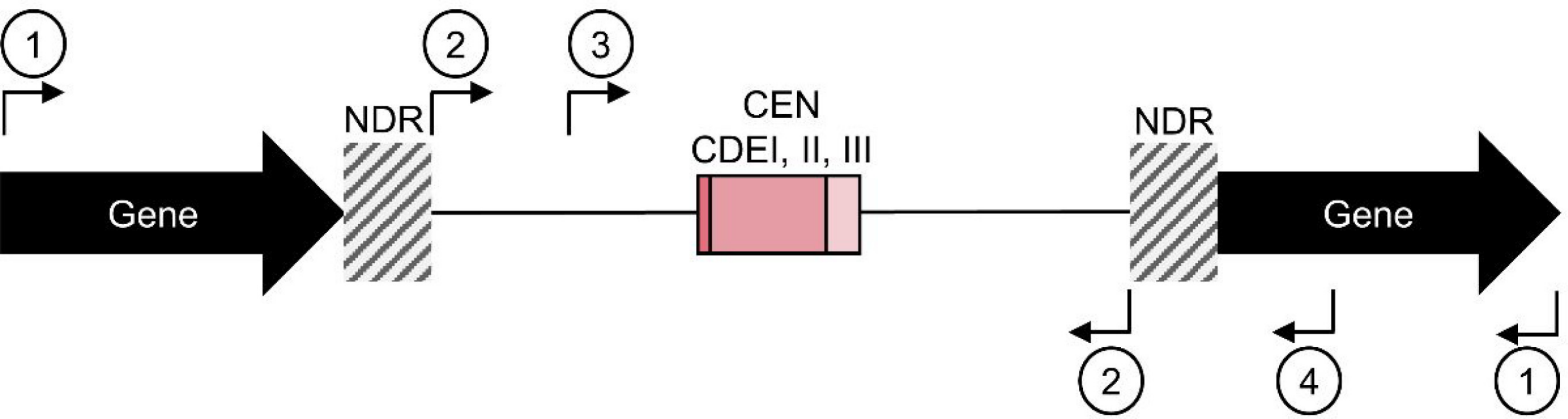				
Centromere	Readthrough from neighboring gene (1)	Adjacent NDR -Promoter or termination site of neighboring gene (2)	Intergenic (3)	Antisense of neighboring gene (4)
**I**		CDEIII-Ent		CDEI-Ent
**II**	CDEIII-Ent	CDEI-Ent		
		CDEIII-Ent		
**III**	CDEI-Ent		CDEIII-Ent	
**IV**	CDEI-Ent	CDEIII-Ent		
	CDEIII-En**t**			
**V**			CDEI-Ent	
			CDEIII-Ent	
**VI**	CDEI-Ent	CDEIII-Ent		
**VII**	CDEI-Ent	CDEIII-Ent		
**VIII**	CDEIII-Ent	CDEIII-Ent		CDEI-Ent
**IX**	CDEI-Ent			
	CDEIII-Ent			
**X**	CDEIII-Ent	CDEI-Ent		
		CDEIII-Ent		
**XI**	CDEI-Ent	CDEI-Ent		
		CDEIII-Ent		
**XII**		CDEI-Ent		
		CDEIII-Ent		
**XIII**	CDEI-Ent	CDEIII-Ent		
**XIV**	CDEI-Ent	CDEIII-Ent		
**XV**		CDEIII-Ent		CDEI-Ent
**XVI**	CDEI-Ent			CDEIII-Ent

### Centromeres are protected from pericentromeric transcription in G1

We next analyzed global transcription at all centromeres and their vicinity by plotting the median number of reads across all 16 of them (Figure 2C). Surprisingly, in G1, most chromosomes display pericentromeric transcription (periCEN) converging towards the centromere that primarily stops within 50 bp of the centromere border (Figure 2C, filled line). Only a small fraction is able to readthrough the CEN where it usually terminates after ∼10–20 bp (Figure 2C, filled line). Indeed, only 1.6% of periCEN reads enter the CEN (Supplemental Figure S3B), consistent with data showing that RNA PolII pauses near the CDEI border and within the CDEIII element in logarithmically grown cells (52). As shown in Figure 2C, both DNA strands are transcribed. We next looked at the prevalence of each transcription orientation, either entry into the centromere through the CDEI or the CDEIII element. During G1, most cenRNAs enter through the CDEIII element (except for *CEN8*) (Supplemental Figure S4A and B), suggesting that the CDEIII border is more permissive to the transcription machinery than the CDEI element (52). During S phase, global pericentromeric transcription increases but most importantly, a higher proportion of this transcription enters the CEN (Figure 2C, dotted lines, 2D and Supplemental Figure S3B). Indeed, the median of the proportion of periCEN transcripts entering the CEN is ∼67% in S phase compared to 6% in G1 (Figure 2D). Interestingly, kinetochores are transiently disassembled during S phase (33) and the active passage of the replication fork was shown to be required for cenRNA production (9). This suggests that kinetochores likely represent a physical barrier to the transcription machinery that is partially alleviated during DNA replication (29). Increased CEN accessibility affects transcription on both strands similarly (Supplemental Figure S4A and B). However, each individual centromere appears to have a preferential direction of transcription (Supplemental Figure S4C). This preference is maintained for most CENs regardless of whether transcription partially or completely covers the CEN sequence (Supplemental Figure S4D). Nonetheless, despite increased CEN accessibility, transcription tends to terminate primarily along the CDEII element (Figure 2C), suggesting that specific cues, like the CENP-A^Cse4^ nucleosome and/or specific DNA elements embedded in CDEII element, may promote transcription termination. The Iso-Seq profiles of *CEN1* and *CEN8* are shown in Figure 2E as representative examples of these various features (see Supplemental Figure S5 for all 16 CENs). We validated these data by qPCR at both *CEN4* and *CEN5*, using specific sets of primers to amplify periCEN or CEN-entering transcripts (cenRNA) (Supplemental Figure S6).

Overall, our Iso-Seq data revealed a previously uncharacterized transcriptional landscape around centromeres. Most pericentromeres are likely transcribed during the whole cell cycle and terminate in the close vicinity of centromeres. It is only during S phase that centromeres become more accessible, and pericentromeric transcripts leak through and become cenRNAs, suggesting that specific factors might be involved in the control of centromere accessibility during the cell cycle.

### Cbf1 represses (peri)CEN expression outside of S phase

**Figure 3.**
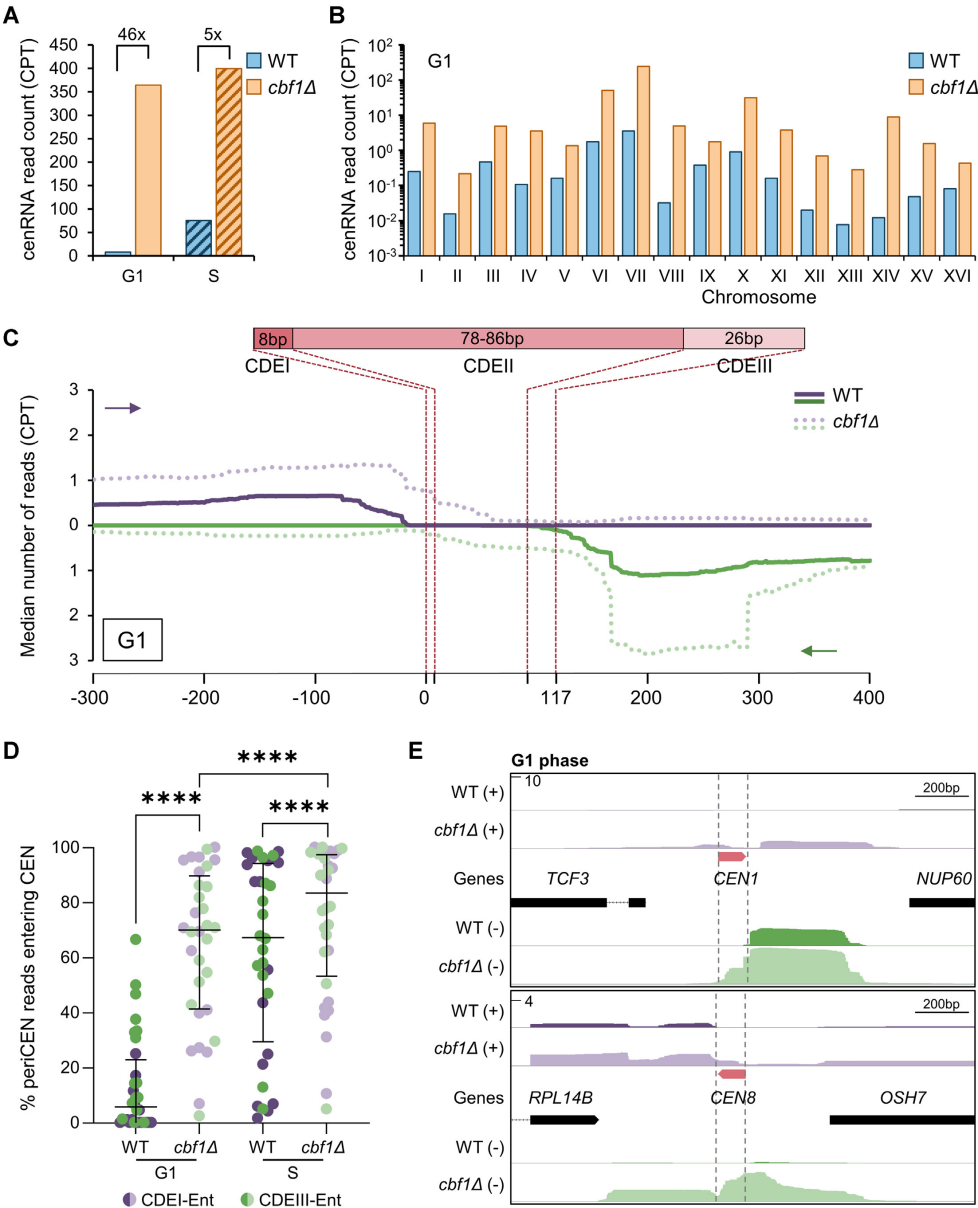
Cbf1 negatively regulates cenRNA expression and promotes transcription termination at the CEN border in G1. (**A**) Total number of normalized cenRNA read counts measured in counts per thousand (CPT) after hybridization capture enrichment. (**B**) Distribution of normalized cenRNA read counts per chromosome. (**C**) Aggregate plot of median read count around centromeres. All 16 centromeres, regardless of the strand of origin, have been aligned at the beginning of the CDEI element (top plot) or the beginning of the CDEIII element (bottom plot). The 5′–3′ direction is indicated by a purple arrow for reads converging toward the CDEI element and a green arrow for reads converging towards the CDEIII element. The structure of the centromere is schematically shown on the top. (**D**) Distribution of the proportion of pericenRNA reads that enter the CEN. Each centromere is represented by two dots, for reads coming from each strand. Median plus interquartile range is displayed in black. *P*-values determined by a paired Wilcoxon test (****P < 0.0001). (**E**) Example of Iso-Seq profiles at *CEN1* and *CEN8* in G1 for WT and *cbf1Δ* cells. Position of centromere boundaries is shown by the vertical grey dotted lines. Reads coming from the (+) strand are shown in purple while reads coming from the (–) strand are shown in green. The y-axis represents the maximum CPT. All WT data are copied from Figure 2 and shown for comparison purposes.

Because of its direct binding to the CDEI element and its repressive role in CEN transcription, Cbf1 is a good candidate to regulate CEN accessibility during the cell cycle (9,25,31). To test this hypothesis, we performed hybridization capture Iso-Seq in *cbf1Δ* cells at G1 and S phase. Consistent with previous observations of increased cenRNA expression in *cbf1Δ* cells (9,31), total sequenced cenRNA reads were increased by 46-fold and 5-fold in *cbf1Δ* G1 and S phase cells respectively, compared to WT (Figure 3A). All 16 centromeres showed an upregulation of transcription in G1 and S phase, although levels varied widely between chromosomes suggesting centromere-specific effects of Cbf1 loss on transcription (Figure 3B and Supplemental Figure S7A), although we cannot exclude hybridization capture variances across samples. Interestingly, the transcription profile of *cbf1Δ* G1 cells was more reminiscent of WT S phase cells than that of WT G1 cells (Supplemental Figure S7A), suggesting that Cbf1 loss could mimic a constitutive S phase transcriptional behavior at centromeres. However, additional mechanisms might be at play during S phase to promote CEN transcription as cenRNAs levels were even higher in *cbf1Δ* cells.

Although the transcription initiation and termination sites of (peri)cenRNAs do not change much between WT and *cbf1Δ* cells (Supplemental Figure S5), the expression levels of these transcripts and the ratio of cenRNA to (peri)cenRNAs vary greatly. Overall, transcription initiated in the pericentromere is increased in G1 in *cbf1Δ* cells compared to G1 WT cells (Figure 3C), although a subset of pericentromeres remains unaffected by Cbf1 loss (Supplemental Figures S5 and S7B). This suggests that Cbf1 directly or indirectly controls transcription initiation of most but not all centromeres. Most importantly, Cbf1 loss drastically increased the accessibility of the centromere to the transcription machinery in G1 (Figure 3C–E) as 60% of periCEN reads entered the CEN by at least 1 bp compared to 1.6% in WT cells (Supplemental Figure S7C). Nonetheless, transcription still tends to terminate within the CDEII element (Figure 3C and E and Supplemental Figure S5), although the proportion of transcripts overlapping the full CEN is increased by 60-fold in G1 in *cbf1Δ* cells compared to WT (Supplemental Figure S7C). Increased CEN accessibility occurs on both DNA strands, although a stronger effect is observed for transcripts entering through the CDEI element, mainly in G1 (Supplemental Figures S7D and S7E). However, the prevalence of one major orientation for each centromere remains overall unaffected (Supplemental Figure S7F). RT-qPCR analysis on *CEN4* and *CEN5* validated the increased accumulation of (peri)cenRNAs throughout the cell cycle in *cbf1Δ* cells (Supplemental Figure S8).

Taken together, these data highlight a key role of Cbf1 in the regulation of both transcription initiation of (peri)cenRNAs and most of all in the accessibility of the centromere to the transcription machinery. Notably, it strongly suggests that Cbf1 could act as a transcriptional roadblock that physically prevents transcription readthrough (52).

### Cbf1 represses CEN transcription via a roadblock mechanism

**Figure 4.**
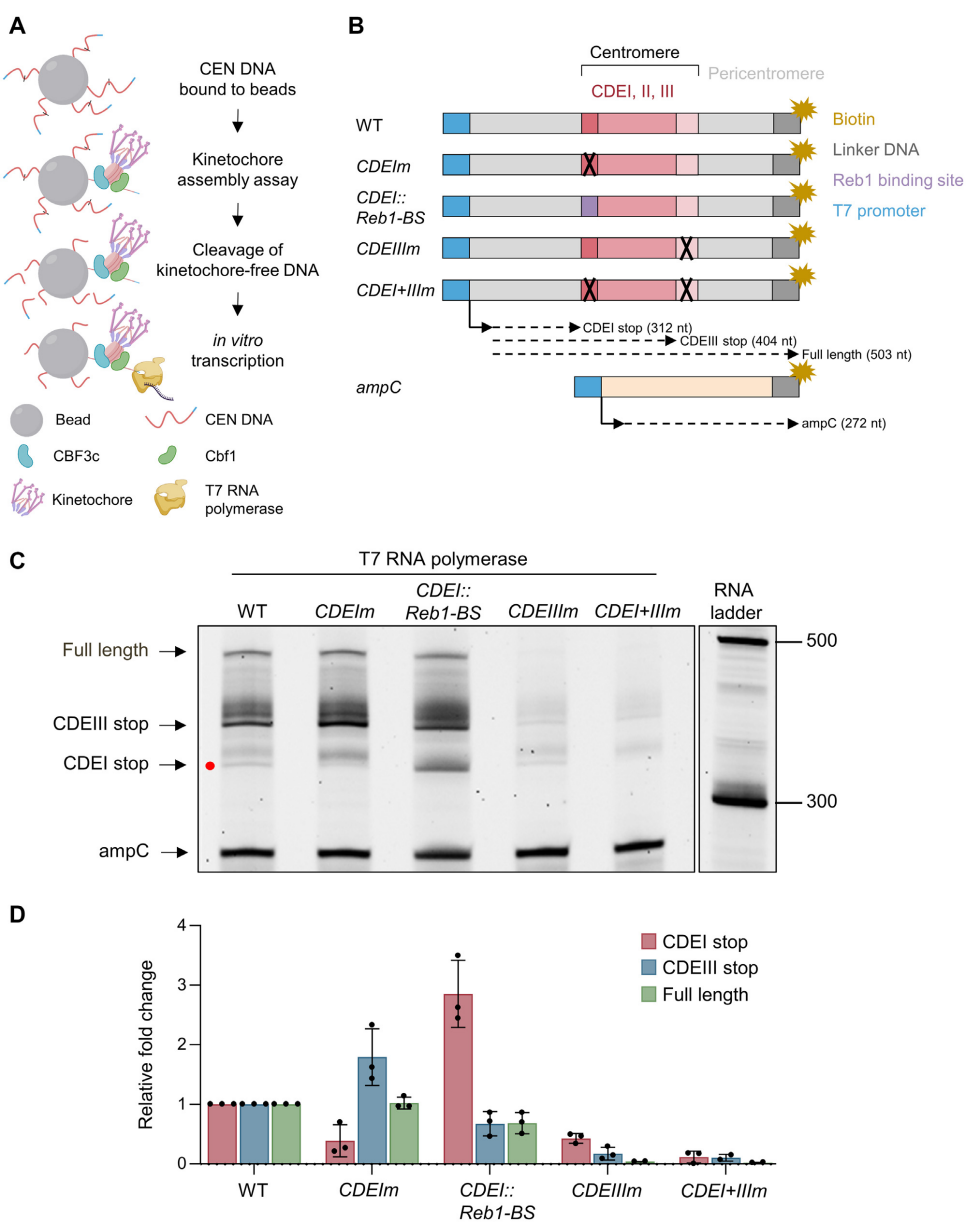
Cbf1 exhibits partial roadblock activity at yeast centromeres *in vitro*. (**A**) Diagram of the combined kinetochore assembly and T7 *in vitro* transcription experiment. First, kinetochores were assembled on the DNA templates presented in (B) and then any kinetochore-free DNA was cleaved by restriction digest. T7 RNA polymerase was added to initiate transcription and the resulting RNA products were purified and analyzed by migration on a TBE-Urea acrylamide gel. Cartoon was created with BioRender.com. (**B**) Schematic of the DNA templates used for the kinetochore assembly assay followed by T7 *in vitro* transcription. The templates include 250 bp from the *E. coli ampC* gene that encodes for β-lactamase (light orange) as an internal control; the 117 bp chromosome III centromere (WT); a mutant *CEN3* (*CDEIm*) containing two point mutations in the CDEI element (black ‘X’) that abrogates Cbf1 binding, a mutant *CEN3* (*CDEI::Reb1-BS*) where the 8bp CDEI element is replaced by the 10bp Reb1 consensus binding site (purple); a mutant *CEN3* (*CDEIIIm*) containing three point mutations in the CDEIII element that abrogates CBF3 complex binding and kinetochore assembly; or a mutant *CEN3* (*CDEI + IIIm*) containing both CDEI and CDEIII point mutations. The three Centromere-Determining Elements (CDEs) are indicated and are flanked by ∼300 bp in 5′ and 70 bp in 3′ of pericentromeric DNA and plasmid backbone (light grey). The DNA templates also contain linker DNA (dark grey) before the biotinylation (dark yellow star) at the 3′ end of the centromere. The CEN template is ∼500 bp. All templates include the 20 bp T7 promoter in 5′ (blue) and the site of transcription initiation is indicated by an arrow. Expected RNA products and associated sizes are indicated by the dashed arrows. (**C**) *In vitro* transcription of CEN DNA after kinetochore assembly. Purified RNA products were separated on a 6% TBE-Urea gel. The major discrete RNA products are indicated by the arrowheads. The band corresponding to the CDEI stop is indicated by a red dot. Sizes are given in nucleotides. Full size gel and negative control where T7 RNA polymerase was omitted is shown in Supplemental Figure S9B. (**D**) Quantification of each major RNA product from (C). Data were first normalized to the ampC signal then to the WT intensity. Error bars represent SD (*n* = 3).

We set out to directly test whether Cbf1 has a roadblock activity at centromeres. Because the disruption of Cbf1 binding to DNA has pleiotropic effects at the chromatin and transcriptional level *in vivo*, we designed an *in vitro* approach to specifically test the roadblock activity. To achieve this, we took advantage of a *de novo* kinetochore assembly assay that promotes assembly of kinetochore proteins on a CEN DNA template (44) and combined it with *in vitro* transcription. Because the T7 transcription polymerase is partially sensitive to DNA roadblocks (57), we added the T7 promoter sequence upstream of the CDEI element to drive T7-mediated transcription after kinetochore assembly (Figure 4A). To perform the assay, *CEN3* DNA templates were bound to beads, incubated with yeast lysates to allow kinetochore assembly, and then briefly incubated with restriction enzymes recognizing restriction sites within the CDEII element to cleave off any kinetochore-free DNA. The resulting beads were then incubated with T7 RNA polymerase and the size of the corresponding transcripts was analyzed (Figure 4A). To test the roadblock activity, we mutated the CDEI element to prevent Cbf1 binding or replaced this element with a 10 bp Reb1 recognition motif since the Reb1 protein is a well-characterized roadblock factor (58) (Figure 4B and Supplemental Figure S9A). As a negative control for the assembly assay, we mutated the CDEIII element alone or in combination with the CDEI mutation (Figure 4B). Finally, to control for the *in vitro* transcription step, CEN DNA beads were mixed with 10% of *E. coli* ampC gene DNA coupled to the T7 promoter sequence (Figure 4B). In the absence of endogenous transcription termination machinery, we expected to observe at least three different RNA isoforms in addition to the control ampC RNA, corresponding to a stop at known DNA binding elements: a short isoform corresponding to a stop at the CDEI element, an intermediate isoform corresponding to a stop at the CDEIII element and a long isoform corresponding to the transcription of the full-length template (Figure 4B). While transcription termination was fuzzy at the CEN, as observed in our Iso-Seq data, we were able to clearly detect these three main isoforms (Figure 4C). Interestingly, the CDEIII element acted as a strong roadblock although a fainter but clear blockage occurred at the CDEI element (Figure 4C and zoomed-in inset in Supplemental Figure S9B). This suggests that Cbf1 may have a weak roadblock activity that can be partially bypassed by the T7 RNA (57). Consistent with this hypothesis, the abrogation of Cbf1 binding to the CDEI element (Supplemental Figure S9A) reduced the production of the short isoform by 60%, concomitantly with a similar increase of blockade at CDEIII element (Figure 4C, D). As expected, Reb1 recruitment to the CEN (Supplemental Figure S9A) led to a 2.7-fold increase of the small isoform (Figure 4C and D). Mutation of the CDEIII element alone or in combination with a CDEI mutation impaired overall assembly (Supplemental Figure S9A), led to subsequent DNA cleavage, and weak T7-mediated transcription (Figure 4C and D). Overall, these data are consistent with Cbf1 partially controlling transcription termination at centromeres.

### Restoring roadblock activity at centromere partially rescues CEN activity

**Figure 5.**
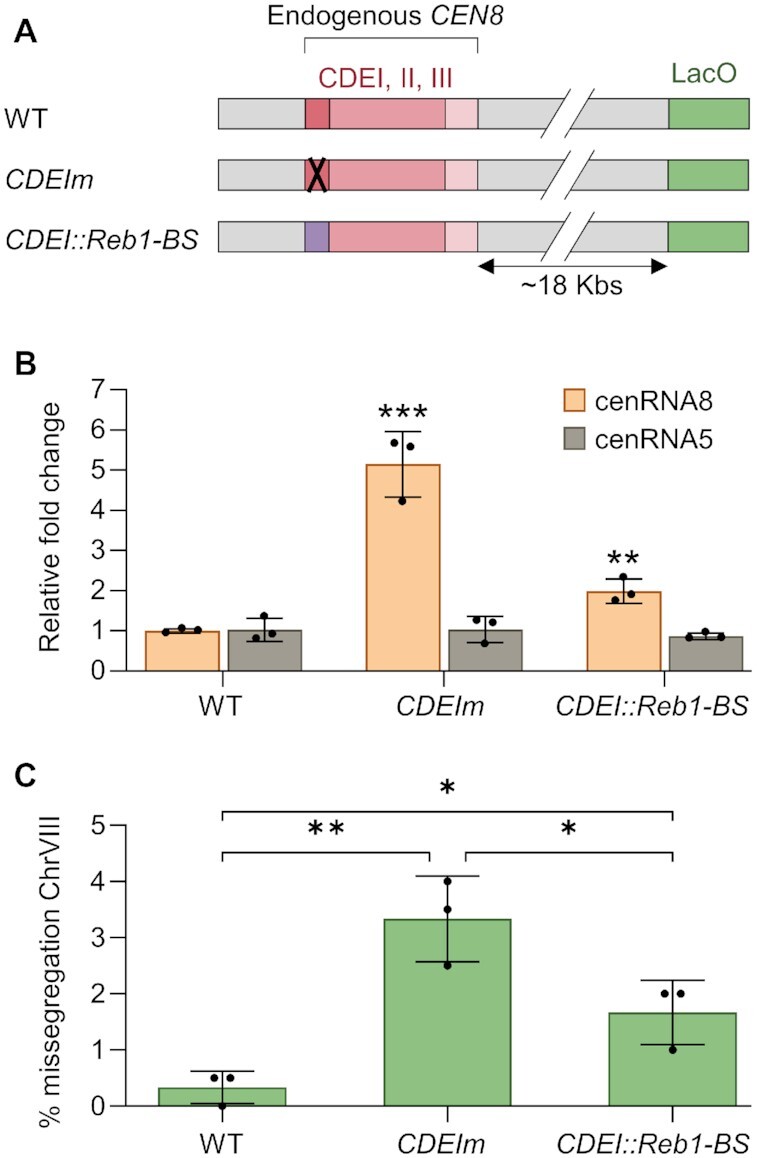
Reb1 recruitment partially restores roadblock activity at centromeres lacking Cbf1. (**A**) Schematic of the mutations introduced on endogenous *CEN8* that is flanked by a LacO array. The mutations on CDEI and the replacement of CDEI by the Reb1 binding motif are identical to those performed on *CEN3* and presented in Figure 4. (**B**) RT-qPCR analysis of cenRNA5 and cenRNA8 expression levels. (**C**) Quantification of chromosome VIII mis-segregation in anaphase (percent of binucleate cells with a fluorescently labeled *CEN8* signal in only one of the two nuclei). Error bars represent SD of three independent experiments; *n* = 200 cells for each experiment. *P*-values for (**B**) and (**C**) were determined using a two-tailed unpaired *t*-test (*P < 0.05; **P < 0.01; ***P < 0.001).

Our Iso-Seq data revealed that, while Cbf1 regulates transcription initiation at a subset of centromeres, it mostly controls the access of the centromere to the transcription machinery via a termination roadblock mechanism. To address if transcriptional roadblock is the main activity of Cbf1 at the CDEI element *in vivo*, we tested whether replacing Cbf1 binding by Reb1 binding could restore CEN transcription levels and activity. Using a CRISPR-Cas9 approach, we introduced point mutations in the CDEI element of *CEN8* to either disrupt Cbf1 binding or to replace CDEI by the Reb1 binding site (same mutations used on the DNA template for the assembly assay in Figure 4) (Figure 5A). Additionally, chromosome VIII was marked with a LacO array to follow its segregation by microscopy. We confirmed the specific lack of binding of Cbf1 and/or Reb1 recruitment to *CEN8* by chromatin immunoprecipitation (Supplemental Figure 10). Since the effect of Cbf1 loss on CEN transcription was most pronounced in G1, we synchronized the cells in G1 with α-factor and analyzed their transcriptional levels. As expected, cenRNA8 levels were increased by 5-fold in the CDEI mutant but returned closer to WT levels when Reb1 was targeted at CDEI (Figure 5B), suggesting that targeting Reb1 at centromeres efficiently restores their low transcriptional activity. Moreover, these mutations on *CEN8* did not affect the transcription of another cenRNA (Figure 5B). We next tested if these mutations would impair the centromere activity of chromosome VIII by tracking its segregation during anaphase. Mutation in CDEI element led to a 3-fold increase of chromosome loss whereas Reb1 recruitment partially rescued this loss, consistent with it restoring roadblock function (Figure 5C). In sum, both our *in vitro* and *in vivo* data suggest that Cbf1 directly promotes a strong transcriptional roadblock at centromeres which contributes to the protection of centromere activity.

## DISCUSSION

Here, we combined long-read RNA isoform sequencing with a probe-based enrichment of lowly abundant RNA species to identify the complete transcriptional landscape at budding yeast centromeres. Because of their very low abundance, most high throughput sequencing datasets lack yeast cenRNAs and they have only been detected by PCR. This has precluded an understanding of their transcription start and stop sites. We overcame this barrier by using the Iso-Seq method combined with hybridization capture to completely sequence the transcripts from the poly(A) tail to the 5′ cap. This approach allowed for the discovery of a complex mixture of transcripts converging towards the CEN from both directions. While we expected to only detect transcription around centromeres in S phase, we surprisingly found that, in G1, most pericentromeres are transcribed, albeit these transcripts stop before the CEN, making cenRNA species a small subset of the surrounding RNA repertoire. This highlights an intriguing heterogeneity between centromeres which, despite harboring very similar sequences, show different accessibility to the transcription machinery and generate different RNA isoforms of varied abundance, suggesting that distinct mechanisms might be at play to regulate CEN transcription during the cell cycle. Another level of heterogeneity is reflected by the absence of well-defined transcription initiation sites, strongly suggesting that cenRNAs are the result of pervasive transcription. Indeed, the surrounding genomic and chromatin environment, which is unique to each centromere, appears to play a key role in that process. Notably, (peri)CEN transcription initiation often relies on promoter or transcription termination sites of flanking genes (Table 1), as observed for most non-coding RNAs (55,56). Interestingly, a few periCEN transcripts overlap with annotated non-coding RNAs (56). However, we did not observe any correlation between cenRNA levels and neighboring gene expression either in G1 or S phase (59). Additionally, local fluctuation in nucleosome positioning might regulate RNA PolII accessibility and transcription towards the CEN, as observed for cenRNAs whose transcription initiation does not depend on neighboring gene elements. In that regard, the loss of the chromatin remodeler Fun30 alters nucleosome phasing around the CENs and results in increased transcription at *CEN3* (60). Additional chromatin remodelers complexes like RSC, ISW2 and SWN/SNF are enriched at CENs and could play a role in the local regulation of chromatin accessibility to the transcription machinery (61–63). Finally, chromatin composition around CENs may add another level of regulation, as observed with the repressive activity of the CEN-adjacent H2AZ^Htz1^ nucleosome or with nucleosomes harboring specific histone point mutations (9,64).

While most converging transcripts stop before the CENs in G1, we found that they partially read through all CENs in S phase, turning into cenRNAs (9,31). Because cenRNAs derive from pericentromeric transcripts, they share the same initiation sites, which remain unchanged in S phase. Despite increased entry into the CEN in S phase, cenRNAs rarely overlap with the entire CEN and unexpectedly tend to terminate within the CDEII element, suggesting that this AT rich element might intrinsically promote transcription termination. Cryptic non-coding transcription termination is regulated by the NNS complex (Nrd1-Nab3-Sen1) which recruits the alternative polyadenylation TRAMP (Trf4/5-Air2/1-Mtr4-polyadenylation) complex to enhance RNA degradation by the nuclear exosome machinery (65). Intriguingly, the NNS complex has a preferential affinity for AU rich motifs (66) and both Nrd1 and Sen1 have been found enriched at centromeres (67). Moreover, mutants of the TRAMP complex or the nuclear exosome machinery accumulate cenRNAs (68,69). Therefore, while the AT richness of CDEII is an important determinant of centromere identity and function, presumably through the maintenance of CENP-A^Cse4^ assembly and stability, we propose that it may additionally promote transcription termination to prevent the disruptive accumulation of transcriptional readthrough. It is currently unclear whether S phase transcription promotes centromere activity or is merely a by-product of chromatin and kinetochore disassembly during DNA replication (33). Interestingly, transcription facilitates CENP-A deposition and maintenance in other eukaryotes (13,17,70). Although yeast kinetochores can assemble *de novo in vitro* in the absence of ongoing transcription, we hypothesize that transcription might facilitate their assembly *in vivo* by opening chromatin to allow CENP-A^Cse4^ deposition or by forming R-loops that have recently been implicated in recruiting kinetochore proteins (71). However, this hypothesis is difficult to test in yeast due to the fast kinetics of assembly (72,73). Nonetheless, our characterization of the centromere transcriptional landscape provides a strong foundation to address these questions in the future.

Altogether, our Iso-Seq data show that the accessibility of the centromere to the transcription machinery is tightly controlled outside of S phase to prevent transcription readthrough. This is consistent with previous proposals that a transcriptional roadblock activity is exerted at centromeres and with data showing that high levels of transcription disrupt kinetochore function (29,30,52,74). Indeed, we found that the transcription factor Cbf1 exerts roadblock activity. Although cenRNA levels are increased in the absence of Cbf1 (9,31), we found its major effect is through the accessibility of the centromere to transcription rather than through the upregulation of total (peri)cenRNA levels. We confirmed this activity *in vitro*, using a reconstituted kinetochore assembly system. Interestingly, despite only binding the CDEI element, Cbf1 regulates CEN accessibility from both the CDEI and CDEIII sides *in vivo*. It is believed that the interaction between Cbf1 and the CBF3 complex stabilizes the Cse4 nucleosome (75,76), so it is likely that Cbf1 loss affects the structural stability of the kinetochore, rendering it vulnerable to the transcription machinery from both directions. To test if transcriptional roadblock is the main activity of Cbf1 at centromeres, we replaced an endogenous CDEI element with the consensus binding sequence of Reb1, a known roadblock termination factor (58). Reb1 recruitment partially rescued both cenRNA levels and the rate of chromosome loss compared to a CDEI mutant, consistent with roadblock activity. Future work should also address whether Cbf1 performs a similar transcriptional roadblock activity at other genomic loci and/or more broadly regulates non-coding transcription. Interestingly, the partial suppression suggests that Cbf1 may also have additional uncharacterized activities at the centromere.

In sum, our study provides the first global analysis of the transcriptional landscape at yeast centromeres, highlighting an unexpected diversity of (peri)cenRNA molecules as well as a novel mechanism regulating centromere accessibility to the transcription machinery. Moreover, we describe a direct transcriptional roadblock activity of Cbf1 at centromeres. Interestingly, a Reb1 binding motif replaces the Cbf1 binding motif at the CDEI element of *sensu lato* saccharomycetes point centromeres (77). Therefore, transcriptional roadblock may be an evolutionary conserved feature of point centromeres to preserve them from detrimental unscheduled transcription. Although Cbf1 binding to centromeres is not conserved, recent studies in human cells reported that the CENP-B DNA binding protein negatively regulates CEN transcription (78,79). An intriguing possibility is that CENP-B provides roadblock function at human centromeres. In the future, it will be interesting to determine if the transcriptional roadblock activity is an evolutionary conserved feature of centromeres that maintains low levels of transcription to promote proper centromere activity.

## DATA AVAILABILITY

Polyadenylation datasets were obtained from (51) under number SRA012232. All sequencing datasets generated in this study have been deposited at the National Center for Biotechnology Information (NCBI) Gene Expression Omnibus (GEO), accession number GSE189063.

## Supplementary Material

gkac117_Supplemental_Files
