# Supplementary material for: A transcriptional roadblock protects yeast centromeres
Source: Nucleic Acids Res. 2022 Mar 7;50(14):7801–15. doi: 10.1093/nar/gkac117 (PMC9371891; doi:10.1093/nar/gkac117)
Supplement: gkac117_Supplemental_Files [file gkac117_supplemental_files.zip › Hedouin_NAR_Supplemental_Data_Revised.pdf]

SUPPLEMENTARY MATERIAL

# A transcriptional roadblock protects yeast centromeres

Sabrine Hedouin, Glennis A. Logsdon, Jason G. Underwood and Sue Biggins

Supplemental Material and Methods

Supplemental Table legends

Supplemental Figures S1-S10

Supplemental note and figure

Supplemental references

## Supplemental Material and Methods

### Immunological methods

To validate the kinetochore assembly assay and the effect of the mutations introduced into the various DNA templates, proteins were eluted from the beads in sample buffer and separated by SDS-PAGE. Proteins were transferred to a nitrocellulose membrane (BioRad, #1620115) and standard immunoblotting was performed. Primary antibodies were used as follows: α-Ndc10 (OD1) 1:5,000 was a generous gift from Arshad Desai; α-Cse4 (9536) 1:5,000 (1); α-Flag (M2, Sigma, #F3165) 1:3,000 ; and α-Myc (9E10, BioLegend, #626802) 1:10,000. HRP conjugated secondary antibodies (GE Healthcare, #NA931 for α-mouse and #NA934 for α-rabbit, both 1:10,000 dilution) were detected with Pierce SuperSignal West Dura enhanced chemiluminescent (ECL) substrate (ThermoFisher Scientific, #PI-34076).

### Chromatin Immunoprecipitation

Chromatin precipitation was performed as described in (2). Briefly, cells expressing Cbf1-Flag and Reb1-Myc in addition to *CEN8* mutations (SBY20916 for WT *CEN8*, SBY20917 for *CDEIm*, SBY20918 for *CDEI::Reb1-BS*, and SBY6456 as an untagged control) were cross-linked for 15 min with 1 % formaldehyde and quenched with 0.125 mM glycine for 5 min at room temperature. Whole-cell lysates were prepared in ChIP lysis buffer (50 mM Hepes-KOH, pH 7.6; 150 mM NaCl; 1 mM EDTA, pH 8.0; 1 % Triton X-100, and 0.1 % sodium deoxycholate) by glass bead beating and sonication to shear the genomic DNA to an average size of 300-500 bp. Immunoprecipitation was performed using ~1.4 mg of chromatin and 20 µL of pre-conjugated protein G Dynabeads (ThermoFisher) to 5 µg of either anti-Flag antibody (M2, Sigma, #F3161) or anti-Myc antibody (9E10, ThermoFisher, #132500), After binding, beads were washed as follows: 2 washes with ChIP lysis buffer, 1 wash with ChIP lysis buffer supplemented with 500 mM NaCl, 1 wash with ChIP wash buffer (10 mM Tris-HCl, pH 8.0; 0.25 M LiCl; 0.5% NP-40; 0.5 % sodium deoxycholate, and 1 mM EDTA, pH 8.0), and 1 wash with TE buffer (10 mM Tris-HCl, pH 8.0, and 1 mM EDTA). Protein-DNA complexes were eluted in 2X Stop buffer (20 mM Tris-HCl, pH 8.0; 100 mM NaCl; 20 mM EDTA, pH 8.0, and 1% SDS) and cross-links were reversed overnight at 65 °C. Eluates were incubated with 4 µg of RNase A/T1 mix (ThermoFisher, #EN0551) for 1 hr at 37 °C, then with 4 U of Proteinase K (NEB, #P8107S) for 3 hr at 37 °C. DNA was recovered by phenol chloroform extraction and ethanol precipitation. qPCR was performed using Forget-Me-Not EvaGreen qPCR Master Mix (Biotium, #31045) with primers listed in Supplemental Table S3. qPCR was performed using a Quantstudio™ 5 Real-Time PCR System (Applied Biosystem). Primer efficiency of each primer pair was evaluated by standard curves with 10-fold serial dilutions of gDNA and is specified in Supplemental Table S3. The percentage of inputs was calculated using the ΔΔCt method.

## SUPPLEMENTAL TABLE LEGENDS

Supplemental Table S1. List of yeast strains used in this study**.** Relevant genotypes of the *Saccharomyces cerevisiae* strains used are listed along with the strain number to reference. All strains are isogenic with W303.

Supplemental Table S2. List of plasmids used in this study. Relevant genes and origins of the plasmids used are listed along with the strain number to reference.

Supplemental Table S3. List of primers used in this study. The sequence and purpose of each primer used is listed.

Supplemental Table S4. List of probes used for hybridization capture. The sequence of each biotinylated oligo, its genomic position, size, strand origin, and Tm is listed.

Figure S1

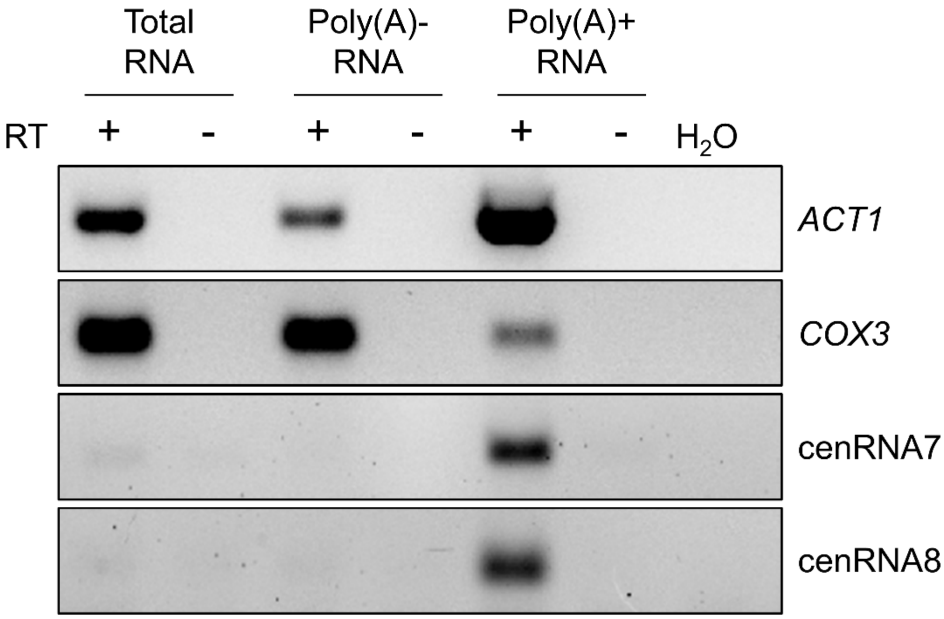


Supplemental Figure S1. CenRNAs are polyadenylated. RT-PCR on cenRNA7 and cenRNA8 using total RNA or RNA fractionated into poly(A) tail enriched (poly(A)+) or depleted (poly(A)-). *COX3*, a mitochondrial mRNA without a poly(A) tail was used as a positive control for the poly(A) depleted RNA fraction while *ACT1* was used as a positive control for the poly(A) enriched RNA fraction.

Figure S2

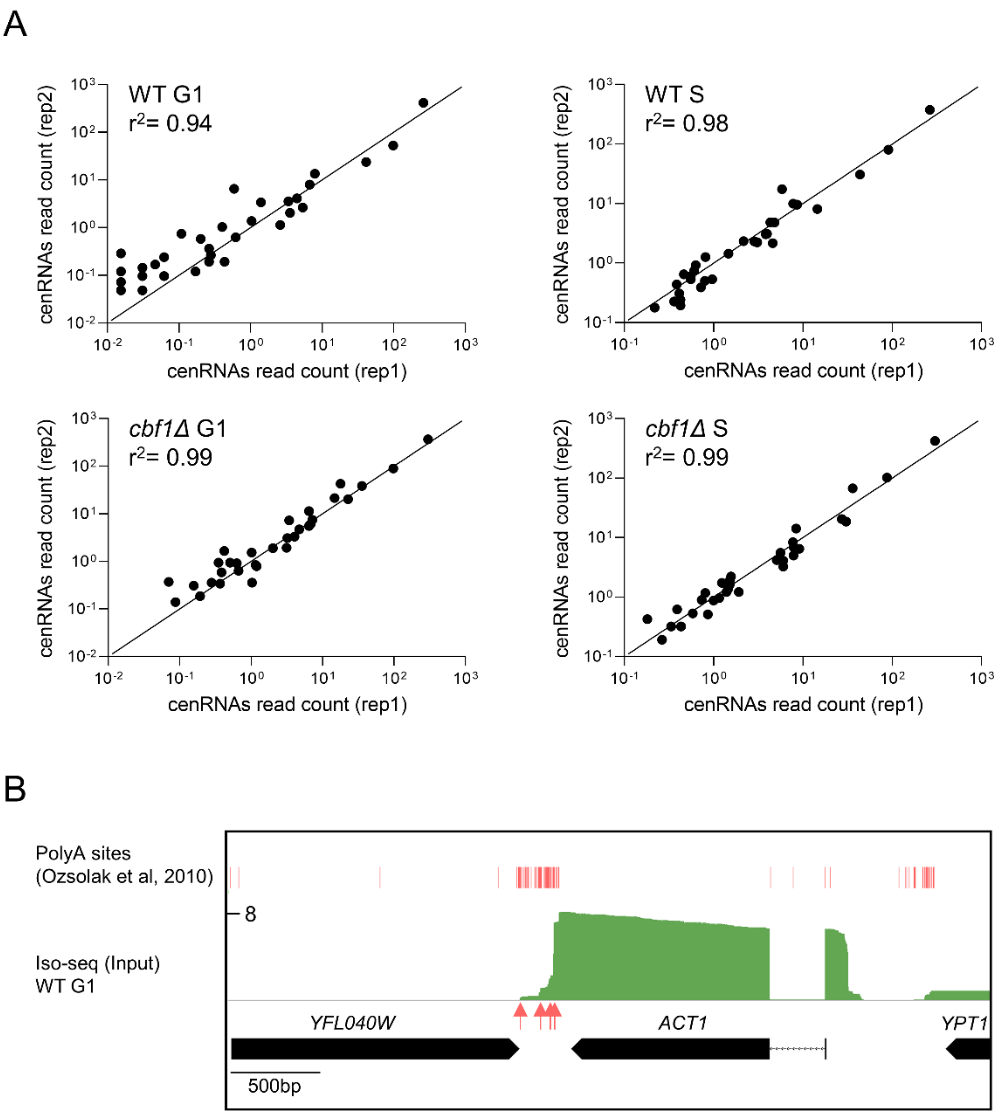


Supplemental Figure S2. Iso-Seq detects complete RNA molecules and alternative polyadenylation sites. (**A**) Pearson correlation analysis of the read count of cenRNAs (in CPT) between the two replicates for each time point and genotype. Each centromere is represented by 2 dots for reads coming from each strand. Not all 32 values are plotted due to the absence of sequenced reads. $r^2$ value and line of identity are displayed on each graph. (**B**) Iso-Seq track example from input data of WT cells in G1 for *ACT1* gene showing the sequencing of full-length RNA with alternative polyadenylation sites (orange arrows) that overlap with previously mapped poly(A) sites (50) (top track).

Figure S3

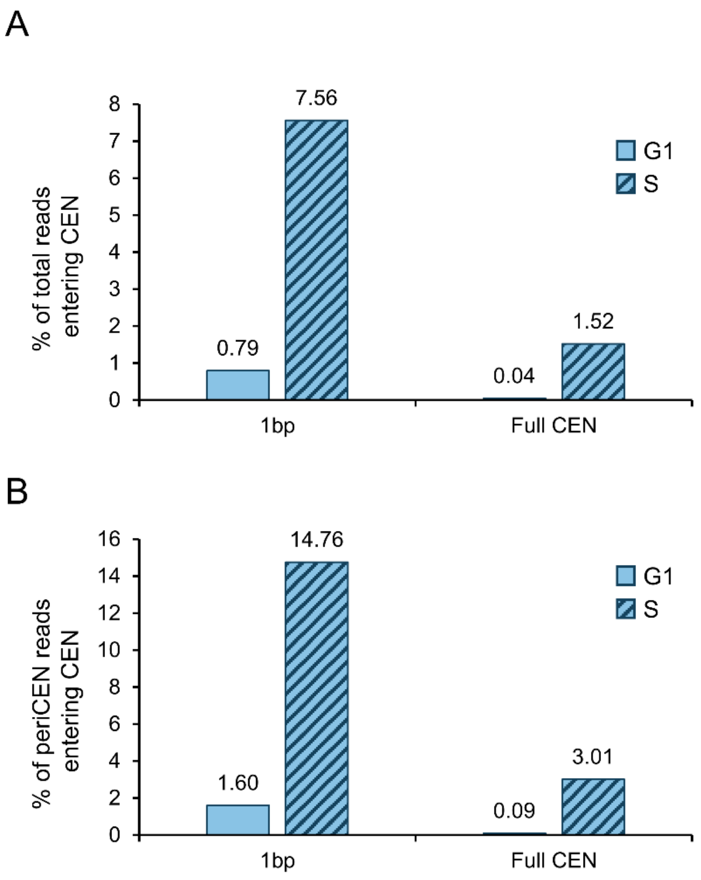


Supplemental Figure S3. cenRNAs represent a small fraction of sequenced transcripts. (**A-B**) Fraction of total sequenced reads (**A**) or of periCEN reads (**B**) that enter the CEN by 1bp, or the entire centromere (full CEN) in G1 and S phase.

Figure S4

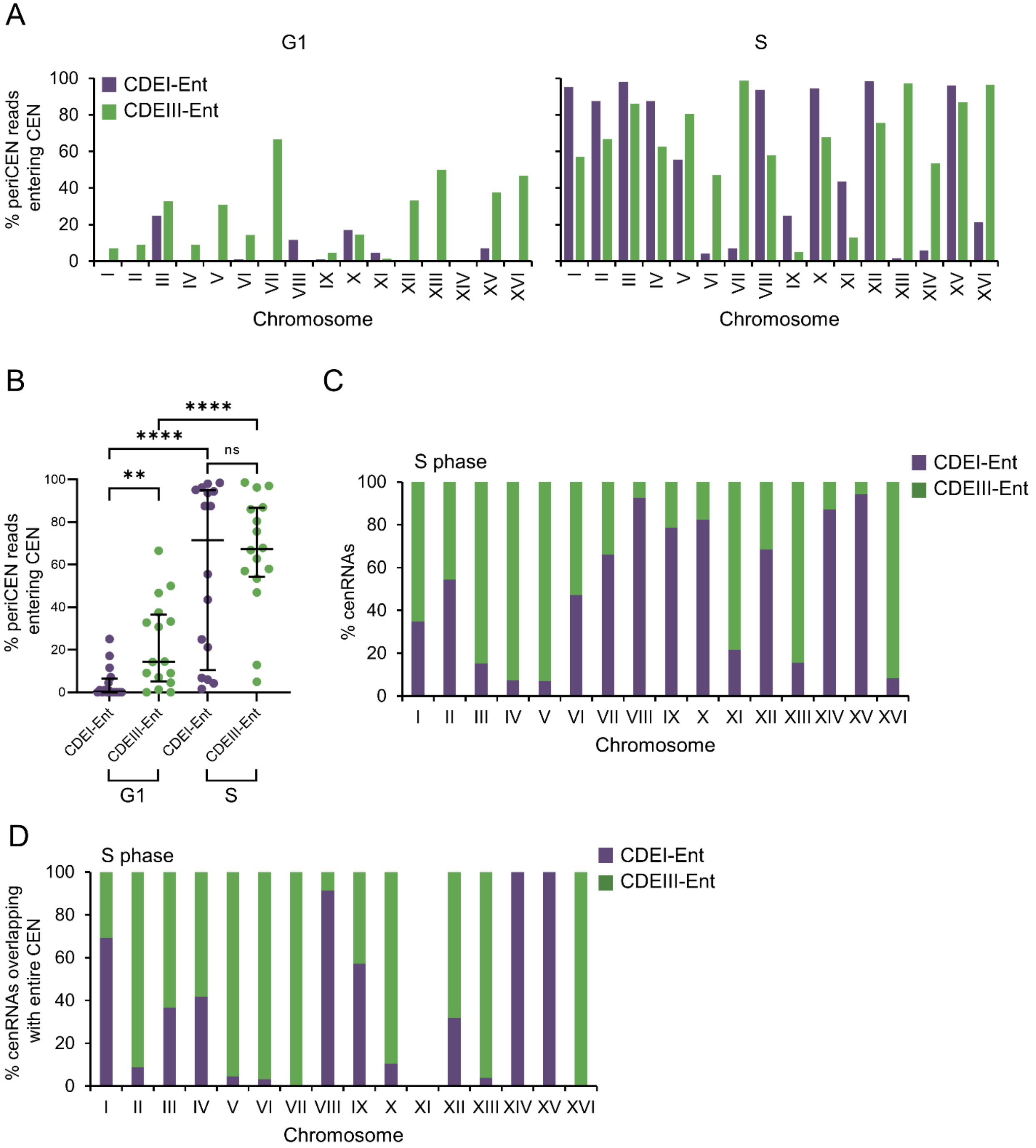


Supplemental Figure S4. Heterogeneity in centromere accessibility. (**A**) Distribution of the proportion of periCEN reads that enter the CEN by chromosome and by transcript orientation (relative to its centromere). (**B**) Proportion of periCEN reads that enter the CEN in G1 vs S phase. Median plus interquartile range is displayed in black. p-values determined by a paired Wilcoxon test (**, $p<0.01$, ****, $p<0.0001$). (**C**) Distribution of the proportion of cenRNA reads by centromere, in S phase, based on their orientation. (**D**) Distribution of the proportion of cenRNA reads that encompass the entire centromere (all three CDEs) by chromosome, in S phase, based on their orientation.

Figure S5

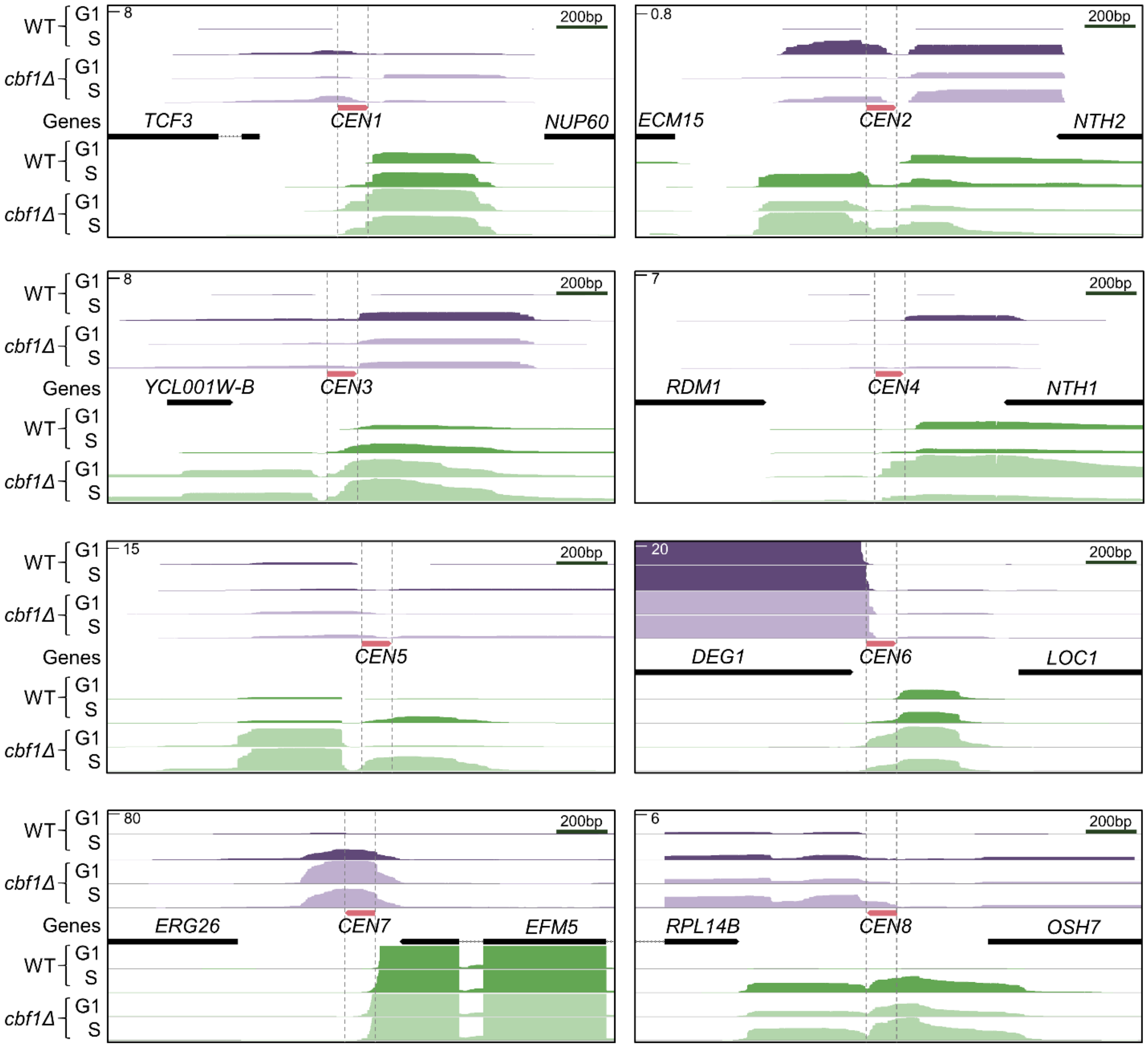

WT
G1
S
cbf1Δ
G1
S
Genes
8
200bp
TCF3
CEN1
NUP60
0.8
ECM15
CEN2
NTH2
YCL001W-B
CEN3
7
RDM1
CEN4
NTH1
15
CEN5
20
DEG1
CEN6
LOC1
80
ERG26
CEN7
EFM5
6
RPL14B
CEN8
OSH7

Figure S5 continued

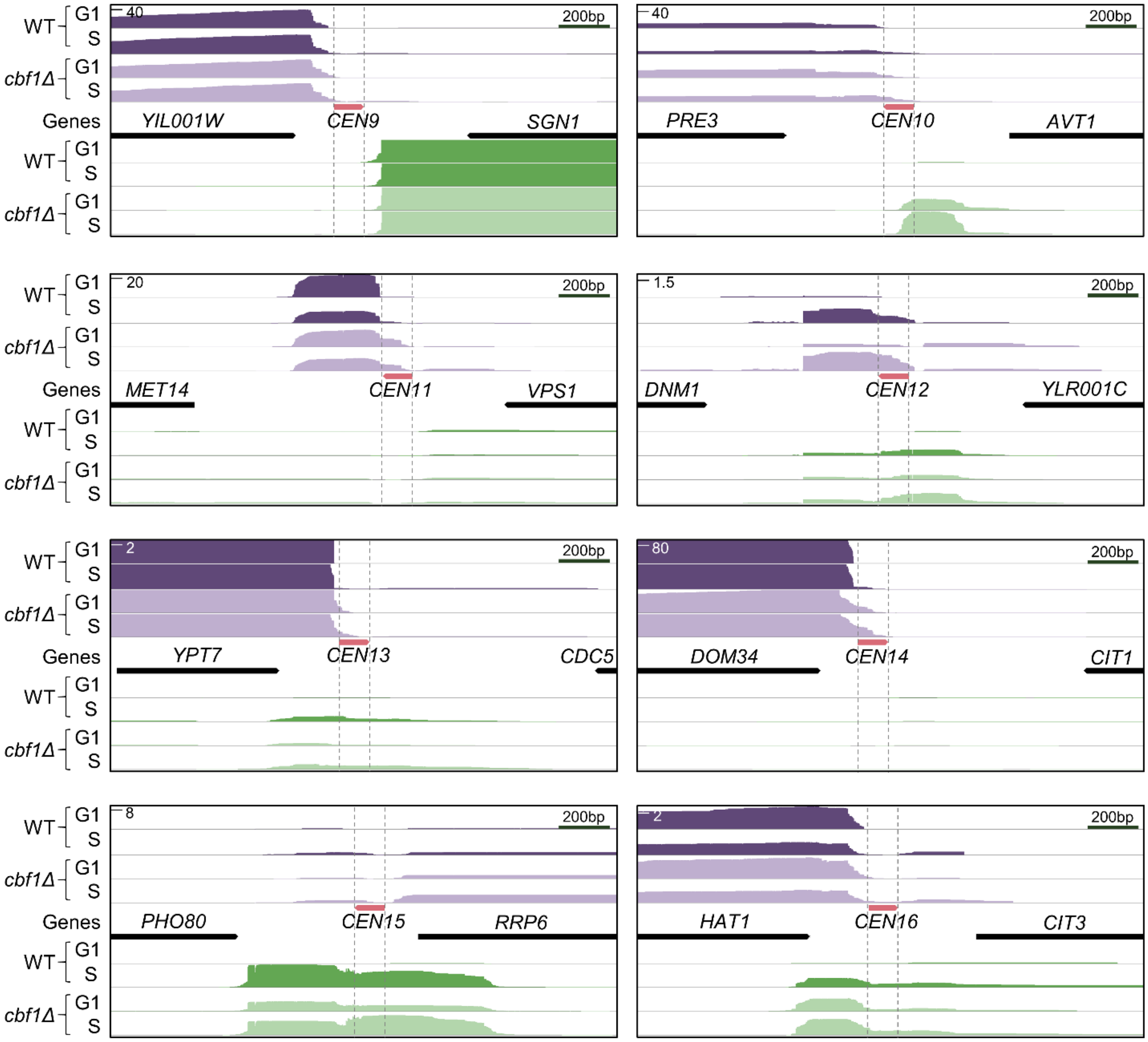


Supplemental Figure S5. Iso-Seq tracks of all centromeres. Screenshots of the Iso-Seq profile around each centromere (pink arrow). Purple tracks and green tracks correspond to reads coming from the (+) strand or (-) strand, respectively. The y-axis is indicated on the left and matches the maximum CPT (except for *CEN6*, *CEN7*, *CEN9*, *CEN13*, *CEN14*, and *CEN16* where it was adjusted to enable visualization of lowly enriched isoforms). Scale bar is indicated on top right corner of each graph.

Figure S6

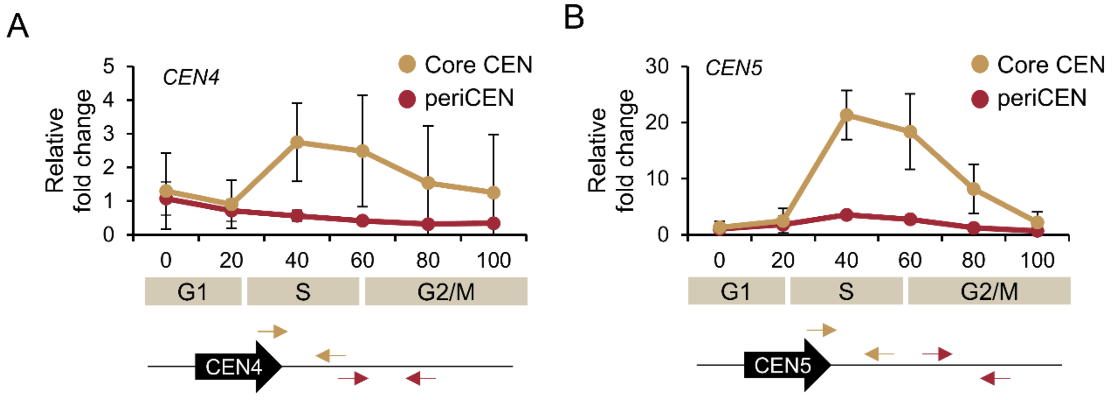


Supplemental Figure S6. Validation of Iso-Seq results. (**A-B**) RT-qPCR analysis of (peri)cenRNA and cenRNA expression of *CEN4* (**A**) and *CEN5* (**B**) during cell cycle progression. Samples were collected at the indicated times after release from α-factor arrest. The approximate timing of cell cycle transitions is indicated beneath the graph. Expression levels were quantified relative to that of t=0 min after α-factor release. A schematic of the position of the primers relative to the CEN is shown below each graph. Error bars represent standard deviation (n=3).

Figure S7

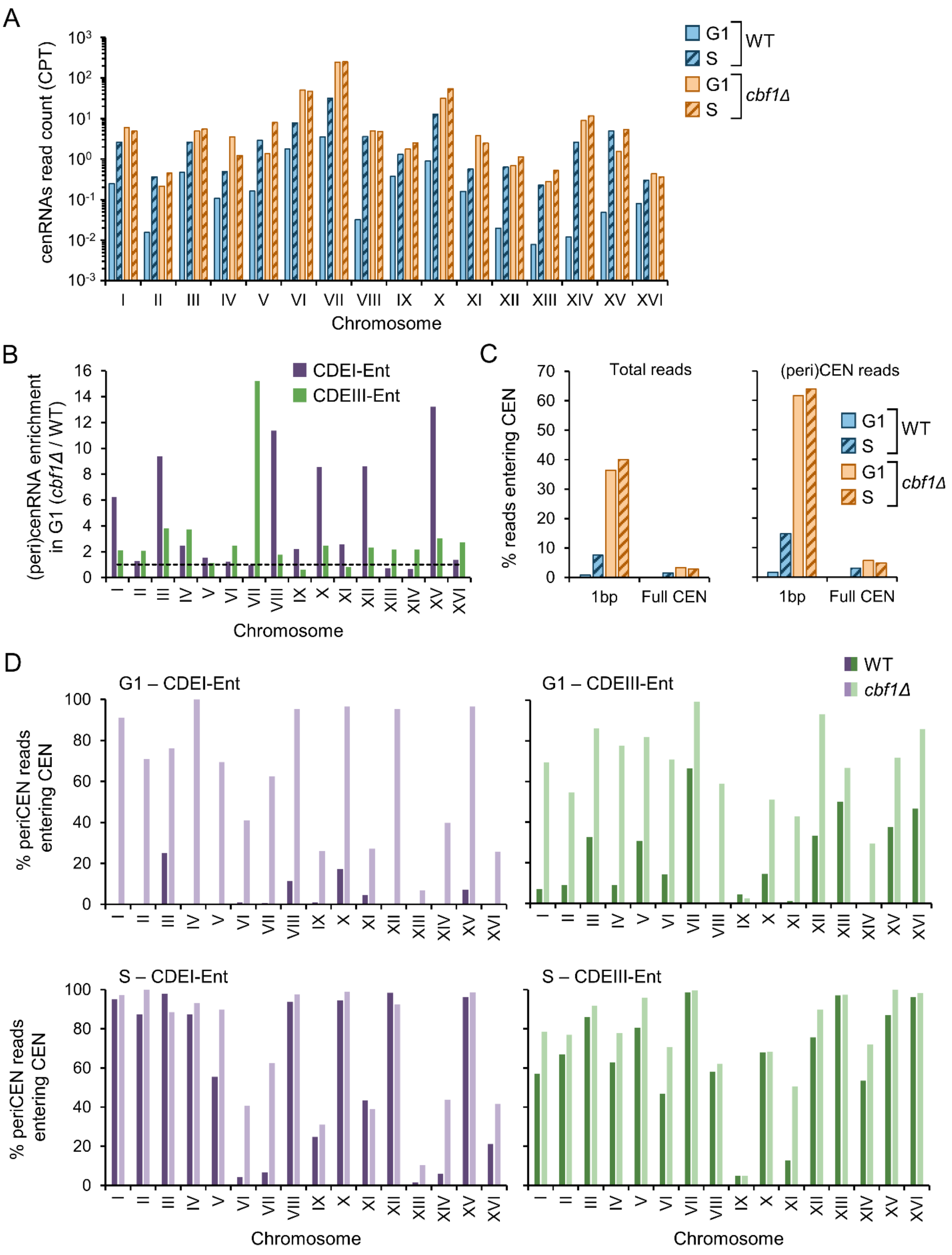

A
cenRNAs read count (CPT)
G1
S
WT
G1
S
cbf1Δ
Chromosome
B
(peri)cenRNA enrichment in G1 (cbf1Δ / WT)
CDEI-Ent
CDEIII-Ent
Chromosome
C
% reads entering CEN
Total reads
(peri)CEN reads
1bp
Full CEN
D
G1 – CDEI-Ent
G1 – CDEIII-Ent
S – CDEI-Ent
S – CDEIII-Ent
WT
cbf1Δ
% periCEN reads entering CEN
Chromosome

Figure S7 continued

E

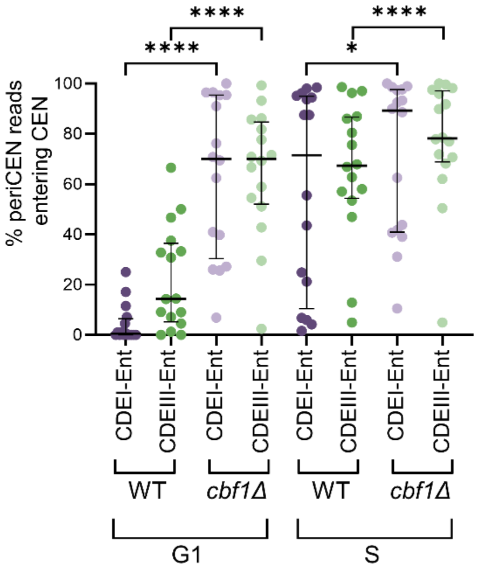


F

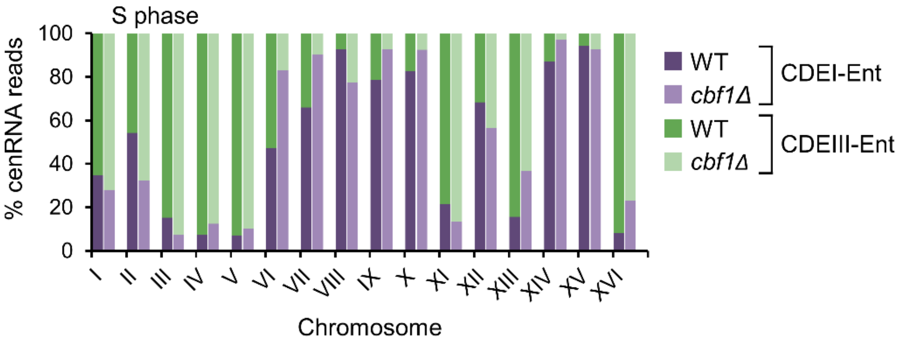


Supplemental Figure S7. Cbf1 loss leads to increased cenRNAs expression through increased accessibility of the centromere domain. (**A**) Distribution of cenRNAs read counts (entering CEN by at least 1nt) per chromosome. (**B**) Distribution of the ratio of total (peri)centromeric reads (periCEN + CEN) in G1 between WT and *cbf1Δ* cells by chromosome and by transcript orientation. The black dotted line represents a ratio of 1. (**C**) Proportion of total reads (left panel) or periCEN reads (right panel) that enter the CEN by 1 bp or the entire centromere. (**D**) Distribution of the fraction of periCEN reads that enter the CEN by chromosome and by transcript orientation in WT and *cbf1Δ* cells. (**E**) Proportion of periCEN reads that enter the CEN in WT vs *cbf1Δ* cells at both cell cycle stages. Median plus interquartile range is displayed in black. p-values determined by a paired Wilcoxon test (*, p<0.05, ****, p<0.0001). (**F**) Distribution of cenRNA reads in S phase according to their transcriptional orientation in WT and *cbf1Δ* cells. All WT data are copied from previous figures and are shown for comparison purposes.

Figure S8

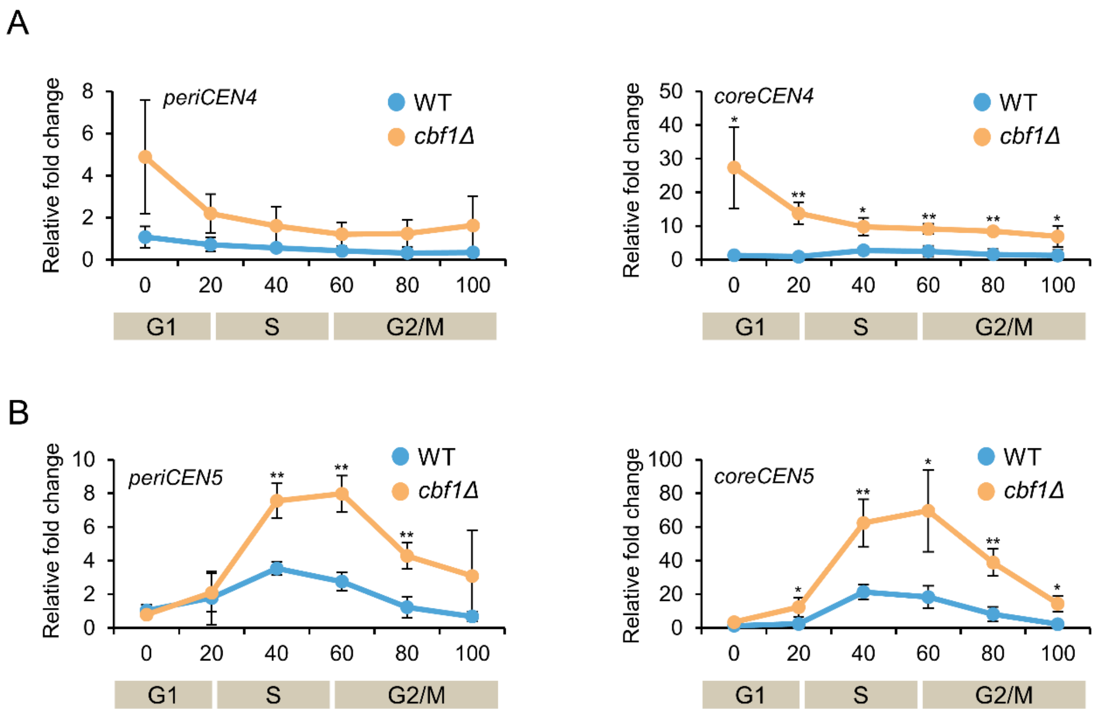


Supplemental Figure S8. Validation of Iso-Seq results in *cbf1Δ* cells. (**A-B**) RT-qPCR analysis of (peri)cenRNA and cenRNA expression of *CEN4* (**A**) and *CEN5* (**B**) during cell cycle progression in WT and *cbf1Δ* cells. Samples were collected at the indicated times after release from α-factor arrest. The approximate timing of cell cycle transitions is indicated beneath the graph. Expression levels were quantified relative to that of WT t=0 min after α-factor release (mean ± SD, n=3). Statistical significances between WT and *cbf1Δ* cells at each time point were analyzed by a two-tailed unpaired t-test (*, $p<0.05$; **, $p<0.01$). The WT data are the same as shown in Supplemental Figure S6.

Figure S9

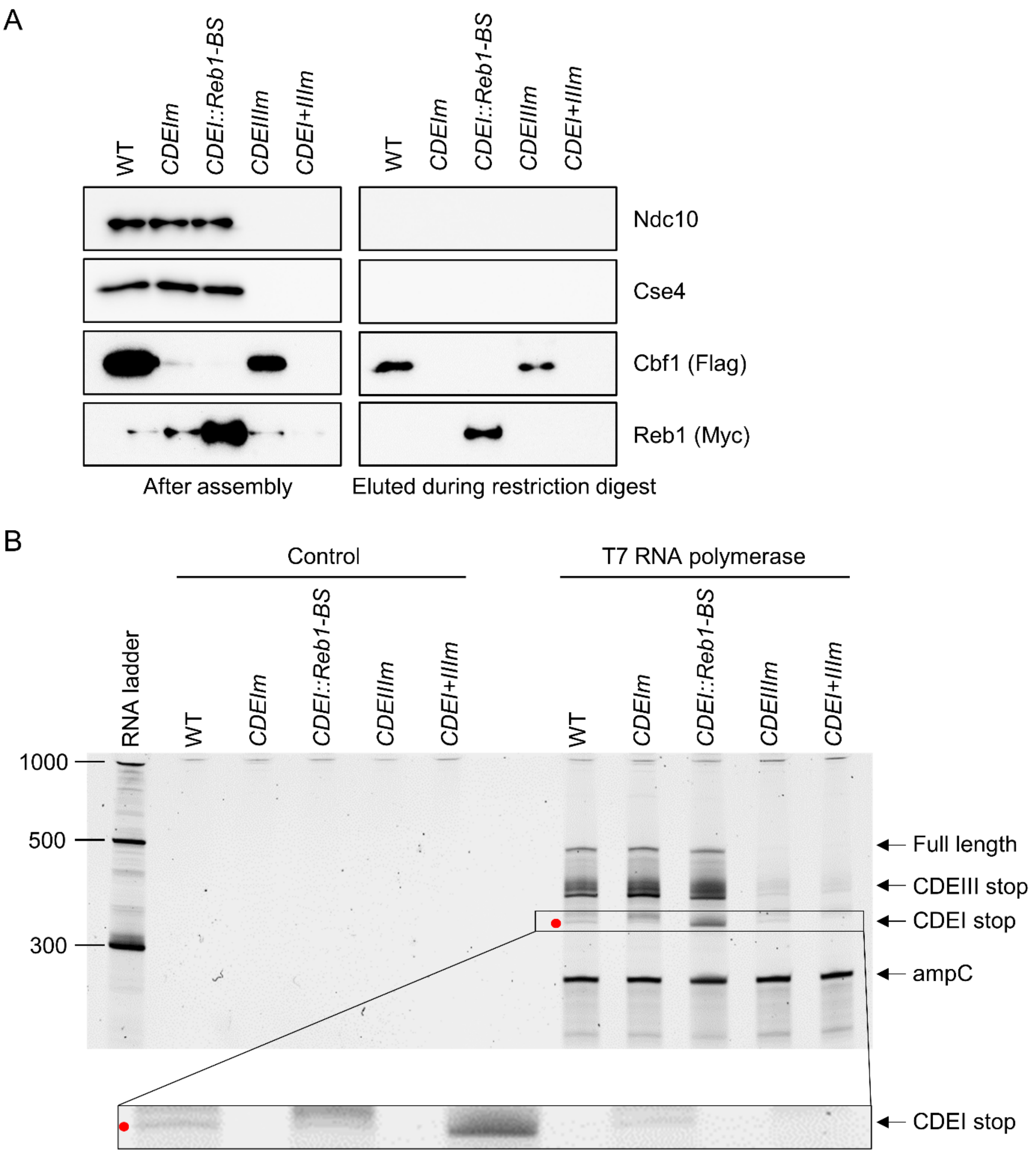


Supplemental Figure S9. Cbf1 has a roadblock activity at centromeres. (**A**) Extract from a CBF1-3xFlag REB1-13xMyc CNN1-3xV5 (SBY19103) strain was used for *de novo* kinetochore assembly assay with the indicated DNA templates. DNA-bound proteins remaining on beads after the initial kinetochore assembly (left) and soluble proteins eluted off during the restriction digest step (right) were analyzed by immunoblotting with the indicated antibodies. We note that the eluted proteins can result from instability on the DNA and/or be proteins associated with cleaved off DNA. (**B**) *De novo* kinetochore assembly was followed by *in vitro* transcription using the T7 promoter and T7 RNA polymerase. Purified RNA products were separated on a 6% TBE-Urea gel. Major discrete RNA products are indicated by the arrowheads. The band corresponding to the CDEI stop is indicated by a red dot and is shown as a zoomed-in inset. Sizes are given in nucleotides**.**

Figure S10

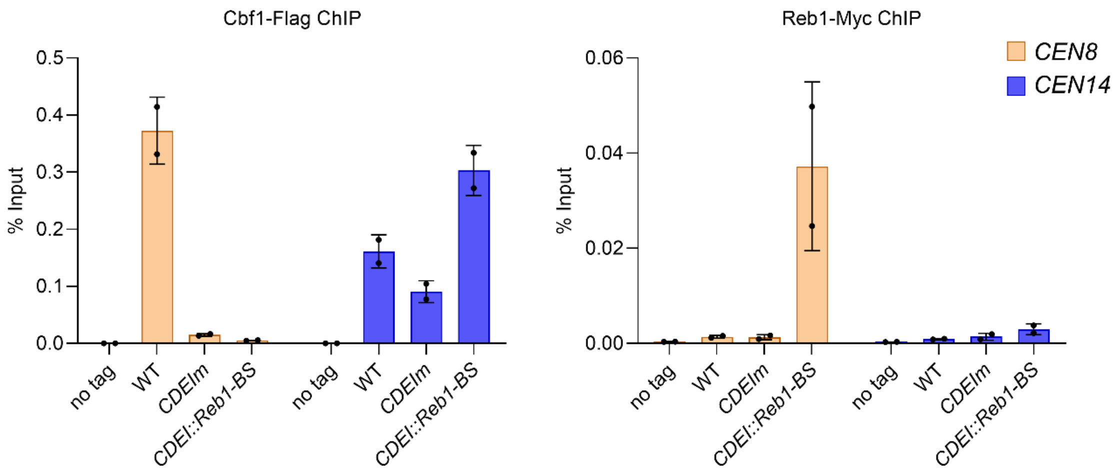


Supplemental Figure S10. Validation of lack of Cbf1 association and Reb1 recruitment to CEN8 *in vivo*. ChIP-qPCR analysis of Cbf1-Flag (left) and Reb1-Myc (right) binding at *CEN8* and *CEN14* in different *CEN8* mutant backgrounds. Error bars represent standard deviation (n = 2).

## Supplemental note

*Divergent transcription*

We surprisingly found that transcription can initiate within the CEN or in its close vicinity and diverge from it (Figure 2C and Supplemental Figure S5). Interestingly, centromeres are flanked by DNase I hypersensitive sites which could be utilized as port of entry for the transcription machinery (3). We quantified this transcription by counting the number of transcripts that start in a region comprising 25 bp of the CEN and 100 bp of flanking DNA (Figure SN-A). Given the compactness of the yeast genome, a few of these divergent transcripts correspond to adjacent gene expression (*CEN11*, *15* and *16*, Supplemental Figure S5), however, the bulk of observed divergent transcripts comes from unannotated non-coding transcription. These transcripts are in very low abundance in G1 and only originate from a small subset of CENs (Figure SNB and C). Similarly, as observed for cenRNAs, divergent transcripts become more enriched in S phase and originate from almost all CENs (Figure SN-B and C), suggesting that the chromatin remodeling occurring during DNA replication at centromeres might facilitate the loading of the transcription machinery. Interestingly, while there is variability between the CENs, transcripts diverging from the CDEIII element are predominant, particularly in S phase (Figure SN-B and D). This correlates with our data showing that the CDEIII border is more permeable to the transcription machinery (Figure 2C).

We next asked whether the loss of Cbf1 would also affect CEN divergent transcription. As observed for convergent CEN transcription, CEN divergent transcripts, from almost all CENs, are highly enriched in a *cbf1*Δ background, in both G1 and S phase, although the levels are variable between CENs (Figure SN-B and C). Noticeably, the proportion of transcripts diverging from CDEI element is more prominent in *cbf1*Δ cells compared to WT cells (Figure SN-D). This suggests that Cbf1 additionally negatively regulates the transcriptional output of CDEI-adjacent pericentromeres. This effect could either be direct or indirect through the regulation of the local nucleosome phasing (4, 5).

Whether CEN-divergent transcripts are just the product of transcriptional noise resulting from altered chromatin architecture or whether they participate in centromere activity is unknown. Recent studies have shown that transcription is required for the proper localization of Shugoshin to the centromere and the maintenance of centromeric cohesion (6, 7). It would be interesting to test if this mechanism is conserved in yeast. Nonetheless, our data shed light on a complex and heterogeneous transcriptional landscape surrounding yeast centromeres during the cell cycle that was previously unappreciated.

Supplementary note

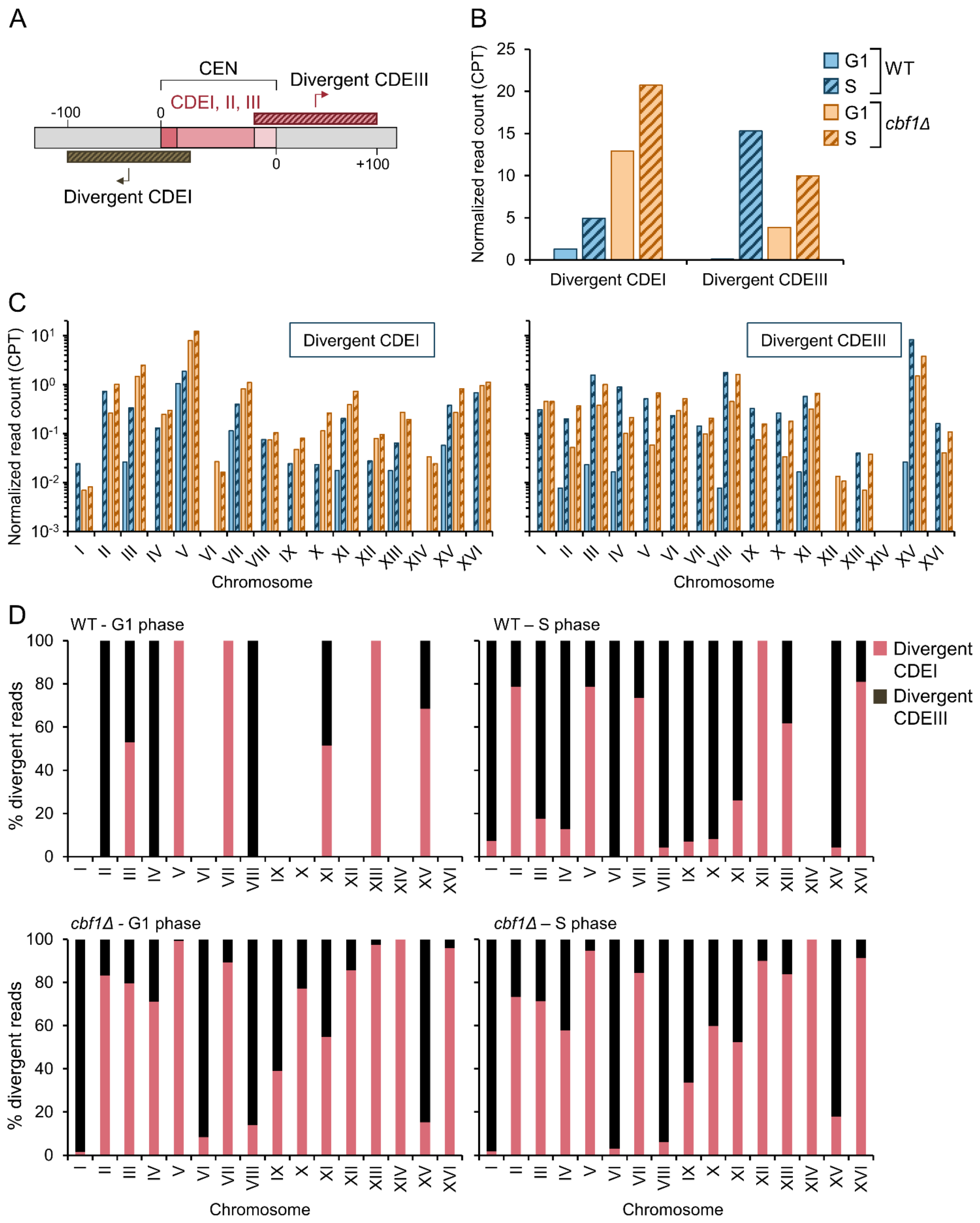

**Supplemental Note. Characterization of the divergent transcription initiating in the vicinity of centromeres.** (**A**) Schematic of the CEN locus. The striped brown and pink boxes represent the regions where starting transcripts diverging away from CDEI or CDEIII element, respectively, were counted. This window corresponds to 100 bp upstream or downstream of the CEN to 25 bp within the CEN. (**B**) Total normalized read count (in count per thousand (CPT)) of CEN divergent transcripts. (**C**) Distribution of normalized read count of CEN divergent transcripts per chromosome. (**D**) Distribution of the proportion of each type of CEN divergent transcript per chromosome.

## Supplemental references


1. Pinsky,B.A., Tatsutani,S.Y., Collins,K.A. and Biggins,S. (2003) An Mtw1 complex promotes kinetochore biorientation that is monitored by the Ipl1/Aurora protein kinase. *Dev. Cell*, **5**, 735–745.

2. Rodriguez,J., McKnight,J.N. and Tsukiyama,T. (2014) Genome-wide analysis of nucleosome positions, occupancy, and accessibility in yeast: nucleosome mapping, high-resolution histone ChIP, and NCAM. *Curr. Protoc. Mol. Biol.*, **108**, 21.28.1-21.28.16.

3. Bloom,K.S. and Carbon,J. (1982) Yeast centromere DNA is in a unique and highly ordered structure in chromosomes and small circular minichromosomes. *Cell*, **29**, 305–317.

4. Saunders,M., Fitzgerald-Hayes,M. and Bloom,K. (1988) Chromatin structure of altered yeast centromeres. *Proc. Natl. Acad. Sci.*, **85**, 175–179.

5. Krassovsky,K., Henikoff,J.G. and Henikoff,S. (2012) Tripartite organization of centromeric chromatin in budding yeast. *Proc. Natl. Acad. Sci.*, **109**, 243–248.

6. Chen,Y., Zhang,Q., Teng,Z. and Liu,H. (2021) Centromeric transcription maintains centromeric cohesion in human cells. *J. Cell Biol.*, **220**.

7. Liu,H., Qu,Q., Warrington,R., Rice,A., Cheng,N. and Yu,H. (2015) Mitotic transcription installs Sgo1 at centromeres to coordinate chromosome segregation. *Mol. Cell*, **59**, 426–436.
